# Collapsed mitochondrial cristae in goat spermatozoa due to mercury result in lethality and compromised motility along with altered kinematic patterns

**DOI:** 10.1038/s41598-020-80235-y

**Published:** 2021-01-12

**Authors:** Bhawna Kushawaha, Rajkumar Singh Yadav, Dilip Kumar Swain, Priyambada Kumari, Akhilesh Kumar, Brijesh Yadav, Mukul Anand, Sarvajeet Yadav, Dipty Singh, Satish Kumar Garg

**Affiliations:** 1 College of Biotechnology, Mathura, India; 2 Department of Veterinary Pharmacology and Toxicology, Mathura, India; 3 Department of Veterinary Physiology, Mathura, India; 4 UP Pandit Deen Dayal Upadhyaya Pashu Chikitsa Vigyan Vishwavidyalaya (Veterinary University), Mathura, 281001 Uttar Pradesh India; 5 ICMR-National Institute for Research in Reproductive Health (NIRRH), Mumbai, India; 6 National Institute of Animal Biotechnology (NIAB), Hyderabad, India

**Keywords:** Apoptosis, Necroptosis, Mechanism of action, Metals, Gene expression analysis

## Abstract

Earlier we have reported mercury-induced alterations in functional dynamics of buck spermatozoa through free radicals-mediated oxidative stress and spontaneous acrosome reaction. Based on our earlier findings, we aimed to investigate the effect of mercury exposure on motility, kinematic patterns, DNA damage, apoptosis and ultra-structural alterations in goat spermatozoa following in vitro exposure to different concentrations (0.031–1.25 µg/ml) of mercuric chloride for 15 min and 3 h. Following exposure of sperm cells to 0.031 µg/ml of mercuric chloride for 3 h, livability and motility of sperms was significantly reduced along with altered kinematic patterns, significant increase in per cent necrotic sperm cells and number of cells showing DNA damage; and this effect was dose- and time-dependent. Contrary to up-regulation of Bax gene after 3 h in control group, there was significant increase in expression of Bcl-2 in mercury-treated groups. Transmission electron microscopy studies revealed rifts and nicks in plasma and acrosomal membrane, mitochondrial sheath, and collapsed mitochondria with loss of helical organization of mitochondria in the middle piece of spermatozoa. Our findings evidently suggest that mercury induces necrosis instead of apoptosis and targets the membrane, acrosome, mid piece of sperms; and the damage to mitochondria seems to be responsible for alterations in functional and kinematic attributes of spermatozoa.

## Introduction

Lead, mercury, cadmium, nickel and arsenic have been reported to adversely affect male fertility including fertilizing competence of spermatozoa^1^. In-vivo toxicological studies have shown that heavy metals have the tendency to accumulate in testis and disrupt endocrine and regenerative capacity of testicular cells^2^. Mercury is extensively used in day-to-day life like in dental amalgam, thermometers, batteries, logical gadgets, electrical switches, semiconductors, additives, pesticides, and pharmaceutical formulations^3,4^. Toxicity of mercury in males, especially its effect on testicular functions, following in vivo exposure of animals has been reported^5–7^. Certain in-vitro studies have revealed adverse effects of mercury on functional dynamics of spermatozoa of bulls^8^, humans^9^, rats^10^, monkeys^11^, and fish^12^. We too have recently reported mercury-induced significant alterations in functional dynamics of goat spermatozoa following in vitro exposure due to significant decrease in total antioxidant defense, increase in intracellular calcium and cAMP levels along with spontaneous acrosomal reaction, inhibition of tyrosine phosphorylation and capacitation like events in sperm cells^13^.

Various pathways of mercury-induced cell death have been reported in different cells^14–18^. Biochemical mechanisms of sperm cell death, especially the genes involved in this process are currently under intense study. Ion-deregulation, particularly Ca^2+^, plays an important role in cell death following either apoptosis or necrosis^19^. In contrast to apoptosis, accidental cell death leading to necrosis with karyorhexis, cell shrinkage, dilatation of the endoplasmic reticulum (ER), swelling of cytosol and condensed mitochondria has also been reported^20^. Necroptosis cell death pathway is mediated by changes in functional status of the mitochondria e.g., change in mitochondrial membrane permeability transition, loss of mitochondrial trans-membrane potential (MTP), ROS generation, Ca^2+^ overload and release of cytochrome-c^15^. Intrinsic variability of semen samples as well as individual variations or differences arising as a result of treatments can be better studied by using computer-assisted sperm analysis (CASA) system that allows simultaneous generation of huge data-sets consisting of kinematic trajectories from thousands of spermatozoa^21^. Therefore, CASA systems have evolved rapidly during the last decade due to major innovations in technology^22^.

In view of the above and also our own recent findings about the mechanism of mercury-induced alterations in functional dynamics of buck-spermatozoa^13^, we envisaged to unravel the precise target-site and mechanism of mercury-induced alterations in functional dynamics and kinematics of goat sperm cells to substantiate our hypothesis. Therefore, the present study was undertaken to investigate mercury-induced alterations in motility and motion kinematics of goat spermatozoa along with ultra-structural changes and mechanism(s) of sperm cell death. Effect of mercury on motility and kinematic patterns will help also us in determining the quality and its suitability for cryopreservation, and also delineating the molecular mechanism(s) of its toxic effects apart from providing some insights in evolving suitable cyto-protective measures to counter mercury-induced cell damage, and improve sperm competence for successful fertilization.

## Material and methods

### Experimental animals

Present study was conducted on six healthy adult male goats (bucks) of Barbari breed aging between two and four years and weighing from 25 to 35 kg. Animals were maintained under standard semi-intensive system of management in goat sheds of the Department of Veterinary Physiology of the Institute. Guidelines of the Committee for the Purpose of Control and Supervision of Experiments on Animals, Govt. of India were followed and the study was undertaken after approval of the experimental protocols by the Institutional Animal Ethics Committee (IAEC) of U.P. Pandit Deen Dayal Upadhyaya Pashu Chikitsa Vigyan Vishwavidyalaya Evam Go Anusandhan Sansthan (DUVASU) (Approval No. 110/IAEC/16).

### Semen collection and dilution

Total ninety semen ejaculates were collected from six different bucks during the months of March to May and September to November using artificial vagina (AV) with graduated semen collection cups. The frequency of semen-collection from each buck was twice a week. Immediately after collection, semen samples were kept in CO_2_ incubator at 37 °C, then processed over thermostatically regulated stage at 37 °C and examined under phase-contrast microscope.

Semen ejaculates of good quality, as manifested by mass motility of ≥ 3, sperm motility of ≥ 80% and abnormal sperms morphology of ≤ 10%, were used for detailed investigation as established earlier in our laboratory^23^ and rest of the semen samples were discarded. Concentration of spermatozoa in semen samples were determined using hemocytometer (Improved Neubauer’s chamber). Semen samples were diluted using semen dilution medium (phosphate buffer solution containing 0.5% glucose and of pH 7) as described earlier^13^ to obtain the final working concentration of 50 × 10^6^ spermatozoa/ml.

### Experimental design

Stock solution (1 mg/ml) of mercuric chloride (HgCl_2_ having 73.89% Hg) was prepared in PBS (pH 7.4). The study was undertaken in four treatment groups using four different concentrations of mercury chloride (0.031, 0.125, 0.25 and 1.25 μg/ml) and one control group (PBS + 0.5% glucose). These concentrations of mercuric chloride were 1/40th, 1/10th, 1/5th and equivalent to the LC_50_ value of HgCl_2_ for goat spermatozoa^13^.

### Effect on livability

To determine the percentage of live spermatozoa in semen samples following in vitro exposure to different concentrations of mercury chloride (0.031, 0.125, 0.25 and 1.25 μg/ml) for different time intervals (15 min, 1 h and 3 h), smears from semen samples of different groups were prepared, air-dried and then stained with Eosin-Nigrosin employing the method of Hancock^24^ as briefly described by us in our recent publication^13^.

### Effect on motility and motion kinematics

Effect of mercuric chloride (0.031, 0.125, 0.25 and 1.25 μg/ml) on motility percentage, percent of total motile cells and rapid progressive motility (%), and kinematic patterns of spermatozoa-curvilinear velocity (VCL; µm s^−1^), straight-line velocity (VSL; µm s^−1^), average path velocity (VAP; µm s^−1^), linearity (LIN; %), straightness (STR; %), beat cross frequency (BCF; Hz), wobble (WOB; %), and maximum amplitude of head lateral displacement (maxALH; µm), compared to the control group, was determined using computer-assisted semen analyser (CASA; Biovis-2000, Version V 4.59, developed by Expert Vision Labs. Pvt. Ltd., Mumbai, India, URL:-http://www.expertvisionlabs.com/BiovisCASA.html), sperm counting chamber, negative phase contrast and 10X objective after different times of in vitro exposure (15 min, 1 h and 3 h) as per the method described by Anand et al.^25^. The CASA system was programmed using algorithm based on the size, shape, and detection of sperm head as follows: Frames/s—60, number of frames acquired—61, max velocity (μm/s) for tracking 150 motility min, curvilinear velocity (VCL; μm/s) > 25 motility min, average path velocity (μm/s) > 10 motility min, straight-line velocity (μm/s) > 1 min, track length (% of frames) 51, aspect 0–99,999, area 2–20, axis (major) 4–20, axis (minor) 2–10, compactness 0–50, perimeter ratio 0–99,999, minimum cell size on major axis 20, minimum cell size on min axis 10, magnification × 10 phase, calibration × (pixels/unit) − 1.905 μ, Y (pixels/unit) 1.905 μ, and size of the image 1280 × 960 pixels (58). Spermatozoa were maintained for up to 3 h at 37 °C in dry bath. 5 μl sample from each diluted semen sample (50 × 10^6^/ml) was loaded in metallic sperm counting chamber with surface graticule of 100 × 0.01 sq mm (Sperm processor, Welcomenagar, India) and six fields from each ejaculate and at each time point were measured, and the average values were computed to minimize the measurement errors.

### Effect on DNA fragmentation

The APO-BrdU TUNEL Assay Kit (A23210, Invitrogen) was used to detect mercury-induced DNA fragmentation in spermatozoa following in vitro exposure to mercuric chloride (0.031, 0.125, 0.25 and 1.25 μg/ml) for 15 min and 3 h. After treatment, semen samples was centrifuged at 1500 rpm for 5 min in DPBS (twice) to remove free mercury from the media by removing the supernatant. DNA fragmentation was assessed as per manufacturer’s instructions. Briefly, 5 ml 1% (w/v) paraformaldehyde in PBS was added to 1–2 × 10^6^ sperm cells (in 0.5 ml of phosphate-buffered saline) and placed on ice for 15 min. Samples were centrifuged for 5 min at 300×*g* and the supernatant were discarded. Cells were washed in 5 ml PBS, and then centrifuged to make the pellet and this step was once again repeated to obtain the pellet of treated-cells. Cells were re-suspended in 0.5 ml of PBS. To this, 5 ml of ice-cold 70% (v/v) ethanol was added and the cells were allowed to stand for a minimum period of 30 min in − 20 °C freezer. Total 50 µl DNA-labelling solution was prepared for each sample by adding 10 µl of the reaction buffer, 0.75 µl of TdT enzyme, 8.0 µl of BrdUTP and 31.25 µl of distilled water in tube and mixed well. Re-suspended the cell pellets of each tube in 50 µl of the DNA-labelling solution. The DNA-labelling reaction was carried out at 22–24 °C overnight. After incubation, 1.0 ml rinse buffer was added to each tube and centrifuged at 300×*g* for 5 min. Supernatant was removed by aspiration and this step was repeated by rinsing the cells with 1.0 ml of rinse buffer. Centrifuged the samples at 300×*g* and removed the supernatant by aspiration. 100 µl of the antibody staining solution for each sample was prepared by mixing 5.0 µl of the Alexa Fluor 488 dye-labelled anti-BrdU antibody with 95 µl of the rinse buffer. Cells pellet was re-suspended in 100 µl of the antibody solution and kept at room temperature for 30 min, and during incubation samples were protected from light. 0.5 ml of propidium iodide, a staining buffer, was added to each sample and the cells were incubated again at room temperature for an additional period of 30 min while protecting from light exposure. At least 400 cells were evaluated under fluorescent microscope within 3 h of completing the staining procedure.

### Effect on apoptosis/necrosis

The standard protocol as described by manufacture of Annexin V-FITC (APOAF, Sigma, USA) kit was followed. Briefly, after treatment of spermatozoa with mercuric chloride, sperm cells were washed twice with DPBS and centrifuged at 1500 rpm for 5 min to remove free mercury, if any, present in the media. Then the cells were re-suspended in 1X binding buffer at a concentration of ~ 1 × 10^6^ cells/ml. 500 µl of the cell’s suspension was transferred to plastic test tube (12 × 75 mm) to which 5 µl of Annexin V-FITC conjugate and 10 µl of propidium-iodide (PI) solution were added. The tubes were incubated at room temperature for exactly 10 min while protecting from light. Same procedure was followed for camptothecin (10 μM) treated group sample, the positive control, as well. Fluorescence of cells was immediately captured using fluorescent microscope (excitation at 400–440 nm and emission at 470 nm using 40× objective). At least 400 cells were counted. Live cells showed no staining either by propidium-iodide solution or by annexin V-FITC conjugate. But the cells which were in early apoptotic process were stained with Annexin V-FITC conjugate while the necrotic cells were stained by propidium-iodide. Annexin V-FITC staining was detected as green fluorescence while propidium iodide staining as red fluorescence.

### Expression of apoptotic and anti-apoptotic genes

After exposure to mercury, semen samples were centrifuged at 2000 rpm for 10 min to remove free mercury present in the media, if any, and then the samples were transferred in DEPC-treated micro-centrifuge tubes (Eppendorf). For lysis of the sperm cells, 1 ml of 0.5% Triton X-100 was added to the pelleted spermatozoa for 10 min and then tubes were briefly vortexed and centrifuged at 3000 rpm for 10 min. After that, pelleted spermatozoa were used for RNA isolation.

#### RNA isolation

RNA extraction was carried out using RNA isolation kit (Gene JET K0871, ThermoFischer Scientific, USA) as per the procedure described by manufacturer and stored at − 80 °C for further analysis. Purity of the total RNA was checked by using Biophotometer (Eppendorf, Germany). RNA samples having A260/A280 values equal to or more than 1.8 were used for cDNA synthesis.

#### Synthesis of cDNA

The cDNA was synthesized from the isolated RNA samples using Revert Aid First Strand cDNA Synthesis Kit (ThermoFischer Scientific, USA) following manufacturer’s procedure in thermal cycler (Bio-Rad). The cDNA obtained was stored at − 20 °C. End point PCR conditions were optimized to amplify the Bax, Bcl-2 and β-actin genes sequences by gradient PCR (Bio-Rad) using Dream Taq PCR master mix (K1071, ThermoFischer Scientific, USA). To amplify the desired genes, the already published primer sequences were used (60). These primers were further aligned by using PRIMER BLAST at NCBI; details of which have been given in Table 1.

**Table 1:** Primers sequences of *Bcl*-2, *Bax* and *β-actin* genes.

Primers sequences	EMBL/reference	Product size (bp)	Annealing temp (°C)
Bcl-2-F 5′-TGCTGCTGTTTCTGCCTACA-3′ Bcl-2-R 5′-GCACTTTTGCATGGGTCAA-3′	NM_001166486.1	143	61
Bax-F 5′-CATGGAGCTGCAGAGGATGA-3′ Bax-R 5′-GTTGAAGTTGCCGTCGGAAA-3′	XM_002701934.1	101	60
β-actin F 5′-AGTTCGCCATGGATGATGA-3′ β-actin R 5′-TGCCGGAGCCGTTGT-3′	Dangi et al.^61^	54	60

#### Real-time PCR

Reaction mixture was prepared by adding 1 μl of cDNA template (100 ng/μl), 1 μl each of the forward and reverse primers (10 pmol), 10 μl of PowerUp SYBR Green qPCR Mix (ThermoFischer Scientific, USA), and 7 μl of nuclease-free water to make up the final volume of 20 μl. Initial denaturation was performed at 95 °C for 10 min, annealing at 61 °C for Bcl-2, 60 °C for Bax and β-actin for 30 s, extension at 72 °C for 30 s for 40 cycles, and 72 °C for 5 min for final extension. Light Cycler 480 (Applied Biosystems, USA) was used to analyse the gene expression. Each gene sample was run in duplicate. Non-template control (NTC) was also run simultaneously. Pipetting was done with sterile DEPC-treated tips without creating bubbles to avoid any interference in reading of fluorescence by the instrument. Confirmation of amplification of the specific sequence was done by using agarose gel electrophoresis. 2.5% agarose was mixed with 30 ml of 1X TAE buffer at 60 V/cm. Gene ruler of 50 bp was electrophoresed in parallel to the amplicons (Figs. S1–S6 Supplementary Data).

### Electron-microscopic studies

After treatment of semen samples with the lowest (0.031 μg/ml) and highest (1.25 μg/ml) used concentrations of mercuric chloride for 15 min and 3 h, semen samples of the control and mercury-treated groups were centrifuged at 1500 rpm for 5 min in DPBS (twice) to remove the free mercury, if any, from the media. Then the pellets were re-suspended in the mixture of 2% paraformaldehyde and 2.5% glutaraldehyde in 0.1 M phosphate buffer (PB; pH 7.4) and kept for 12 h at 4 °C for fixation. Then the samples were again cen**t**rifuged at 1500 rpm for 5 min and the supernatant was discarded to remove the fixative. The pellet was suspended in 0.1 M PB, and again centrifuged. Thereafter, the samples (pellets) were fixed for 1 h in 1% osmium tetroxide at 40 °C and then dehydrated in acetone, infiltrated and embedded in araldite CY 212 (TAAB, UK). Sections (0.5 µm) were cut with an ultra-microtome, mounted on to the glass slides, stained with aqueous toluidine blue and observed under light microscope for gross observation of the area and quality of the tissue fixation.

For scanning electron microscopy, after fixation of samples as mentioned above, the samples were centrifuged at 1000 rpm for 10 min and supernatant was discarded. The pellet was suspended in buffer, centrifuged and washed. It was re-suspended in buffer and a drop of it was spread on a cover slip. The samples were air-dried, sputter-coated with colloidal gold and observed under an EVO 18 Zeiss scanning electron microscope at an operating voltage of 20 kV. Images were digitally acquired by using the SmartSEM software.

Thin sections of grey-silver colour interference (70–80 nm thick) were cut and mounted onto 300 mesh copper. The sections were stained with uranyl acetate and alkaline lead citrate, then gently washed with distilled water and observed at Sophisticated Analytical Instrumentation Facility (SAIF), All India Institute of Medical Sciences, New Delhi under Tecnai G2 20 S-Twin transmission electron microscope (Fei Company, The Netherlands) at an operating voltage of 200 kV. Images were digitally acquired by using camera and Digital Micrograph software attached to the microscope.

### Statistical analysis

Data generated were subjected to two-way ANOVA and Tukey’s test using SPSS Version 23.0 where p value of 0 < 0.05 was considered significant. Data presented in tables are mean ± SE of the observations on semen samples of six different male goats. Separate multiple linear regression models were used to examine the effect of different concentrations of mercury and times of exposure on each of the CASA parameters after controlling for potential confounding factors. All the values for kinematic variables were standardized to avoid any scale effect. Correlation was also calculated between the motility and other kinematic patterns.

Analysis of the relative mRNA expression data between different groups was based on the crossing point (Cp) values. The Cp value of each gene was subtracted from the arithmetic mean of Cp value of the β-actin gene to calculate the ΔCt. The relative expression of PCR product was determined by the Eq. (2) (− ΔΔCt) as per Livak method^26^ and the level of significance was set at 0.01% (p < 0.01).

### Ethics committee approval

All experiments involved non-invasive procedures on animals including on large animals are approved by the Institutional Animal Ethics Committee (IAEC) of U.P. Pandit Deen Dayal Upadhyaya Pashu Chikitsa Vigyan Vishwavidyalaya Evam Go Anusandhan Sansthan (DUVASU) as per the guidelines of Committee for the Purpose of Control and Supervision of Experiments on Animals (CPCSEA) of Govt of India. (Approval No. 110/IAEC/16, dated: 16-09-2016).

## Results

### Effect on live spermatozoa count

**Figure 1:**
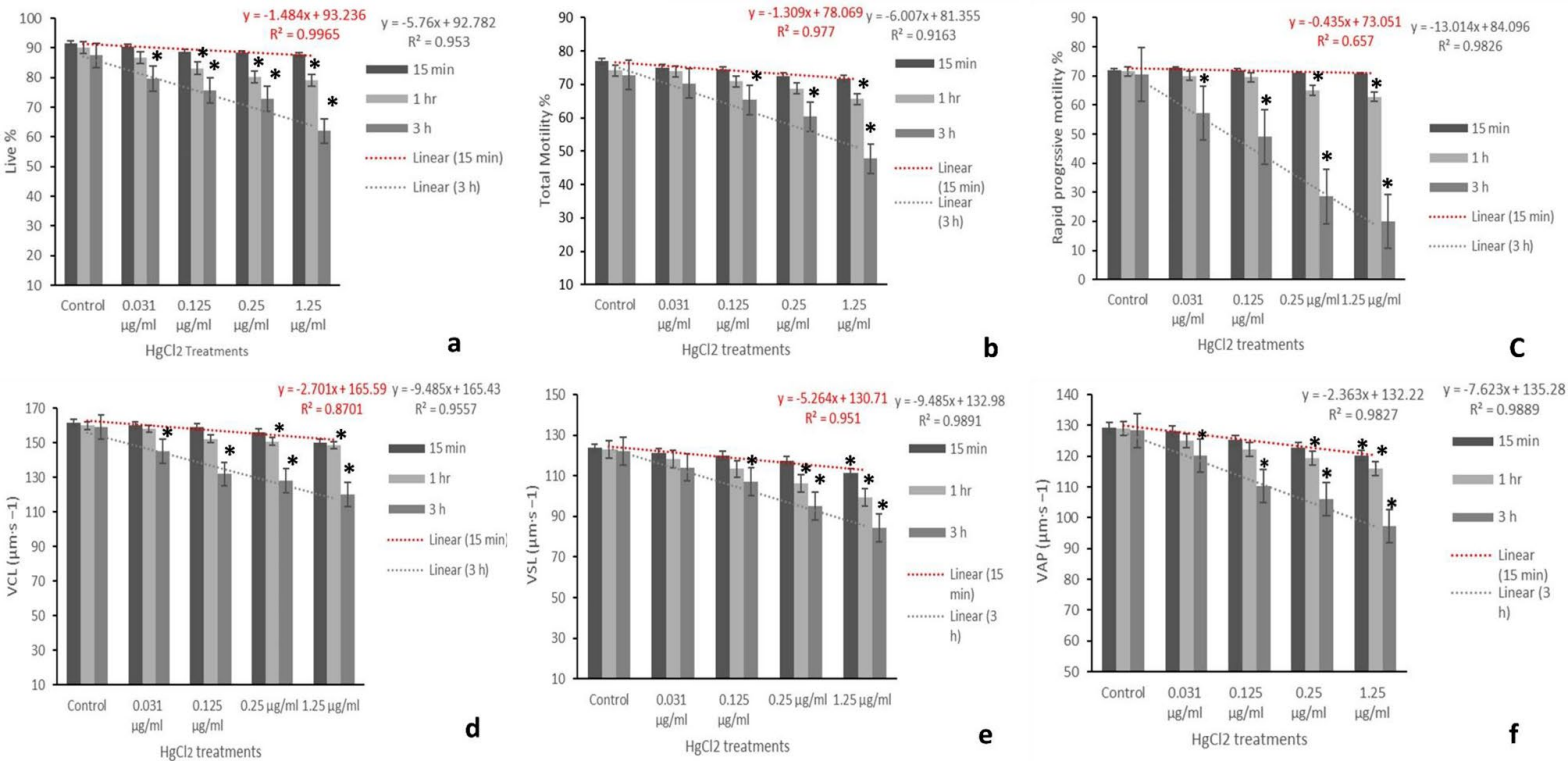
The relationship between different concentrations of HgCl_2_ and computer-assisted semen analysis (CASA) kinematic parameters for per cent live (**a**), per cent total motility (**b**), per cent rapid progressive (**C**), VCL (**d**), VSL (**e**), VAP (**f**). Linear regression lines are shown along with the coefficients of determination (*R*^2^) for 15 min and 3 h. Each data point represents the mean ± SE value of repeated measurements from six male goats.

Compared to the control, there were no significant alterations in per cent count of the live spermatozoa following exposure to different concentrations of mercuric chloride (0.031, 0.125, 0.25 and 1.25 μg/ml) for 15 min. But compared to the control (90.07%), significant (p < 0.05) decrease in live spermatozoa percentage count (80.20 and 79.14%) was observed in semen samples exposed to higher concentrations (0.25 and 1.25 µg/ml) of HgCl_2_ after 1 h. After 3 h of exposure to different concentrations of mercury also, compared to the control (85.44%), significant (p < 0.05) reduction in percentage counts of the live spermatozoa (75.65, 72.82, 65.62%) were observed in groups treated with 0.125, 0.25 and 1.25 µg/ml HgCl_2_, respectively (Table 2, Fig. 1a).

**Table 2:** Effect of *in-vitro* exposure of spermatozoa to different concentrations of mercuric chloride (0.031–1.25 µg/ml) on live spermatozoa count at different time intervals.

Treatments	% live spermatozoa at different time intervals		
	15 min	1 h	3 h
Control	91.61 ± 2.59^A^	90.07 ± 1.97^aA^	85.44 ± 1.66^aA^
0.031 µg/ml mercuric chloride	90.49 ± 1.37^A^	86.71 ± 1.78^abA^	79.58 ± 2.20^abB^
0.125 µg/ml mercuric chloride	88.72 ± 1.36^A^	83.13 ± 1.73^abAB^	75.65 ± 0.83^bB^
0.25 µg/ml mercuric chloride	87.33 ± 1.85^A^	80.20 ± 1.67^bA^	72.82 ± 1.38^bB^
1.25 µg/ml mercuric chloride	85.77 ± 1.27^A^	79.14 ± 1.43^bAB^	65.62 ± 0.04^cB^

Data presented are mean ± SE of the semen samples of six bucks.

Different capital superscripts in the rows indicate significant (p < 0.05) differences between the different time intervals.

Different small superscripts in the columns indicate significant (p < 0.05) differences between the different treatment groups.

### Effect on total motility and progressive motility

Data presented in Fig. 1b,c and Table 3 revealed that compared to the control, there were no significant (p > 0.05) differences in the percentage count of total and progressive motility of spermatozoa in the semen samples exposed to different concentrations of HgCl_2_ after 15 min. But significant (p < 0.05) decrease in total and progressive motile spermatozoa percentage count was observed after 1 h in 1.25 µg/ml HgCl_2_-treated group (65.79 and 58.78%, respectively) compared to the control group after 1 h (84.47 and 71.53%, respectively). After 3 h of exposure to HgCl_2_ at 0.25 and 1.25 µg/ml, there was further decrease in count of the total motile and progressive spermatozoa as these values were found to be 39.84 and 19.9%, respectively.

**Table 3:** Effect of in-vitro exposure of spermatozoa to different concentrations of mercuric chloride (0.031–1.25 µg/ml) on motile spermatozoa count at different time intervals.

	Motility	Rapid progressive	Slow progressive	Non-progressive	VCL	VAP	VSL	LIN%	STR%	WOB%	BCF	maxALH
**15 min**												
Control	85.94 ± 1.2^a^	74.98 ± 0.16^a^	2.23 ± 0.33^a^	6.72 ± 1.45^a^	161.69 ± 0.23^a^	129.26 ± 0.17^a^	123.6 ± 0.61^a^	77.44 ± 0.53^a^	95.62 ± 1.36^ab^	80.74 ± 0.45	41.27 ± 0.31^a^	3.8 ± 0.28^a^
A	88.50 ± 1.2^a^	78.39 ± 1.26^a^	2.96 ± 0.27^a^	6.96 ± 1.23^a^	160.08 ± 0.51^a^	128.21 ± 0.48^a^	121.3 ± 0.42^a^	78.46 ± 1.30^a^	94.61 ± 1.32^ab^	81.51 ± 0.82	40.26 ± 0.44^a^	3.7 ± 0.22^a^
B	86.95 ± 0.5^a^	78.18 ± 1.22^a^	2.82 ± 0.61^a^	4.08 ± 1.7^a^	159.32 ± 0.61^a^	125.18 ± 1.29^ab^	120.02 ± 1.03^a^	77.21 ± 1.40^a^	93.68 ± 1.44^a^	81.42 ± 1.48	39.67 ± 1.11^ab^	3.51 ± 0.41^a^
C	85.62 ± 0.6^a^	76.20 ± 0.72^a^	3.40 ± 0.39^a^	6.48 ± 1.02^a^	156.25 ± 1.71^ab^	122.9 ± 0.36^b^	117.52 ± 1.20^a^	76.28 ± 1.58^a^	94.04 ± 1.03^ab^	82.14 ± 1.20	37.28 ± 0.49^ab^	3.3 ± 0.20^a^
D	84.75 ± 0.3^ab^	74.42 ± 0.81^a^	2.48 ± 0.61^a^	6.44 ± 1.31^a^	150.11 ± 1.43^bc^	120.1 ± 0.81^b^	111.17 ± 1.25^b^	78.01 ± 1.20^a^	95.07 ± 1.05^ab^	82.74 ± 1.06	34.25 ± 0.55^b^	2.91 ± 0.11^ab^
**1 h**												
Control	84.47 ± 1.7^a^	71.53 ± 0.27^ab^	2.22 ± 0.29^a^	7.47 ± 1.03^a^	160 ± 0.38^a^	129.03 ± 1.49^a^	123.03 ± 1.04^a^	77.275 ± 1.06^a^	95.34 ± 1.30^ab^	80.64 ± 1.72	40.52 ± 0.31^a^	3.8 ± 0.21^a^
A	78.06 ± 0.3^ab^	68.02 ± 0.41^b^	4.21 ± 1.37^ab^	6.24 ± 1.36^a^	158.13 ± 1.45^a^	125.1 ± 1.48^a^	118.27 ± 0.36^a^	78.76 ± 0.91^a^	94.34 ± 1.20^ab^	81.47 ± 1.25	39.01 ± 0.32^ab^	3.2 ± 0.22^a^
B	77.73 ± 1.2^b^	67.55 ± 1.59^b^	7.36 ± 1.82^ab^	7.81 ± 1.05^a^	152.45 ± 1.62^b^	122.11 ± 1.72^b^	113.31 ± 0.47^b^	79.58 ± 1.22^a^	93.33 ± 1.03^a^	80.53 ± 1.34	39.08 ± 0.33^ab^	2.81 ± 0.10^a^
C	72.73 ± 1.0^b^	65.04 ± 0.61^b^	5.23 ± 2.21^ab^	8.18 ± 1.51^a^	150.94 ± 0.91^bc^	119.42 ± 1.04^b^	106.16 ± 0.62^c^	80.03 ± 1.42^a^	94.7 ± 1.20^ab^	82.04 ± 1.44	36.28 ± 0.29^ab^	2.52 ± 0.31^ab^
D	65.79 ± 0.4^c^	58.78 ± 0.39^c^	2.38 ± 1.40^a^	9.20 ± 0.91^a^	148.7 ± 0.79^bc^	116.08 ± 1.81^c^	99.32 ± 0.82^d^	80.21 ± 1.39^a^	95.37 ± 1.25^ab^	83.06 ± 1.53	33.28 ± 0.71^b^	2.4 ± 0.13^ab^
**3 h**												
Control	83.61 ± 0.12^a^	70.57 ± 0.18^ab^	4.46 ± 0.41^ab^	8.54 ± 1.61^a^	159.14 ± 1.28^a^	128.31 ± 0.15^a^	122.17 ± 0.41^a^	76.06 ± 1.04^a^	95.12 ± 1.7^ab^	80.27 ± 0.88	39.74 ± 0.48^ab^	3.7 ± 0.29^a^
A	68.23 ± 1.16^c^	52.29 ± 1.26^c^	9.86 ± 1.6^b^	10.78 ± 1.29^a^	145.28 ± 1.32^c^	120.17 ± 1.51^b^	114.07 ± 1.48^b^	77.03 ± 1.42^a^	97.3 ± 1.5^ab^	82.46 ± 0.94	36.04 ± 0.29^ab^	2.8 ± 0.21^a^
B	63.56 ± 1.11^c^	39.02 ± 1.51^d^	20.59 ± 0.26^c^	19.07 ± 1.36^b^	132.02 ± 2.01^d^	110.28 ± 1.04^d^	107.06 ± 1.61^c^	78.85 ± 0.81^a^	97.19 ± 1.07^ab^	82.53 ± 1.37	35.24 ± 0.71^b^	2.4 ± 0.28^ab^
C	47.78 ± 0.31^d^	28.49 ± 1.21^e^	24.67 ± 1.30^ cd^	28.55 ± 1.37^c^	128.17 ± 1.41^d^	106.02 ± 1.01^d^	95.06 ± 1.03^d^	80.92 ± 1.48^a^	98.62 ± 1.40^b^	83.41 ± 1.40	27.93 ± 0.66^c^	2 ± 0.41^ab^
D	39.84 ± 0.28^e^	19.9 ± 0.82f.	29.62 ± 1.7^d^	41.31 ± 1.49^d^	120.27 ± 0.27^e^	97.27 ± 1.06^e^	84.25 ± 0.62^e^	81.19 ± 1.71^a^	98.51 ± 1.03^b^	84.37 ± 1.29	20.61 ± 0.10^d^	1.7 ± 0.66
p-value	0.005	0.0001	0.0123	0.0432	0.0031	0.004	0.022	0.714	0.041	0.844	0.041	0.022

Data presented are mean ± SE of the semen samples of six bucks.

*VCL* curvilinear velocity (µm s^−1^), *VSL* straight-line velocity (µm s^−1^), *VAP* average path velocity (µm s^−1^), *LIN* linearity of forward progression (%), *STR* straightness (%), *WOB* wobble (%), *maxALH* maximum amplitude of lateral head displacement (µm), *BCF* beat-cross frequency (Hz).

A = 0.031 µg/ml, B = 0.125 µg/ml, C = 0.25 µg/ml, and D = 1.25 µg/ml HgCl_2_.

Different small superscripts in the columns indicate significant (p < 0.05) differences in kinematic patterns between control and different treatment groups at different time intervals.

### Effect on kinematic patterns

**Figure 2:**
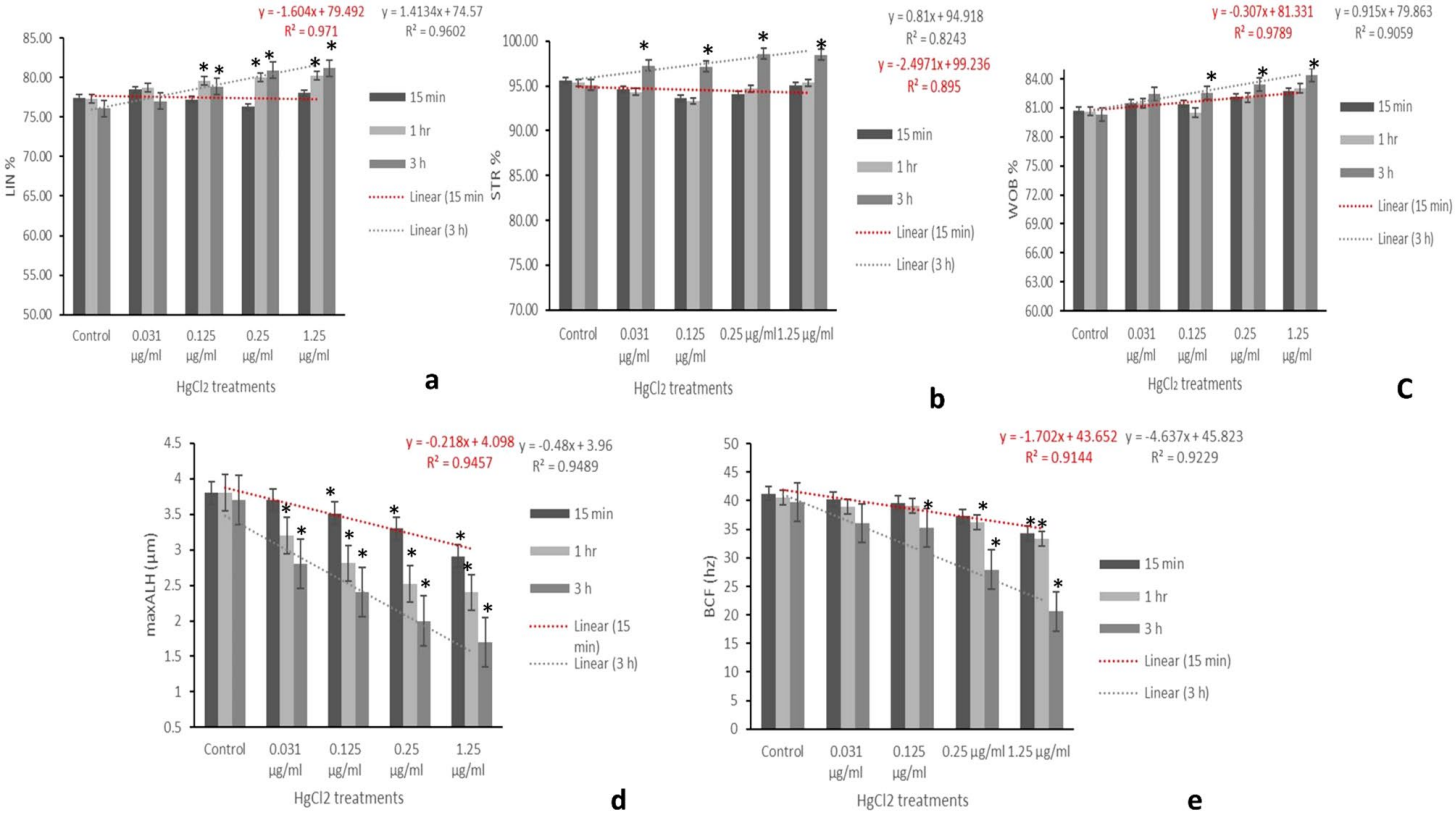
The relationship between different concentrations of HgCl_2_ and computer-assisted semen analysis (CASA) kinematic parameters for per cent linearity (LIN; (**a**)), per cent straightness (STR: (**b**)), per cent WOB (**c**), maximum amplitude of head lateral displacement (maxALH; µm; (**d**)), and beat cross frequency (BCF; hz; (**e**)). Linear regression lines are shown along with the coefficients of determination (*R*^2^) for 15 min and 3 h. Each data point represents the mean ± SE value of repeated measurements from six male goats.

Data presented in Table 3 revealed significant differences in kinematic patterns of the spermatozoa in semen samples treated with different concentrations of mercuric chloride (0.031, 0.125, 0.25 and 1.25 μg/ml) compared to the control group. Compared to the control group after 1 h, slow and non-progressive motility in semen samples treated with different concentrations of mercury were found to be significantly (p < 0.05) increased. However, slow and non-progressive motility were found to significantly (p < 0.05) increase in dose-dependent manner compared to control after 3 h. Different velocity parameters of spermatozoa (VCL, VSL, and VAP) were significantly (p < 0.05) decreased in concentration- and time dependent manner (Fig. 1d–f). Data presented in Fig. 2a–c showed significant (p < 0.05) increase in values of LIN and STR after 3 h exposure to different concentration of mercury as compared to the control group. WOB per cent count was also significantly (p < 0.05) increased as the time of exposure to 0.125, 0.25 and 1.25 µg/ml of HgCl_2_ increased. This suggests that LIN, STR and WOB of spermatozoa were increased and the progressive motility and different velocity parameters (VCL, VAP & VSL) were decreased with increase in exposure time and concentration of mercury; thus, indicating that mercury-induced alterations in sperm progressiveness had relatively greater effect on all the kinematic patterns. However, dose- and time-dependent significant (p < 0.05) decrease in maxALH and BCF, compared to the control group, were observed for up to 3 h (Fig. 2d,e).

### DNA damage

**Figure 3:**
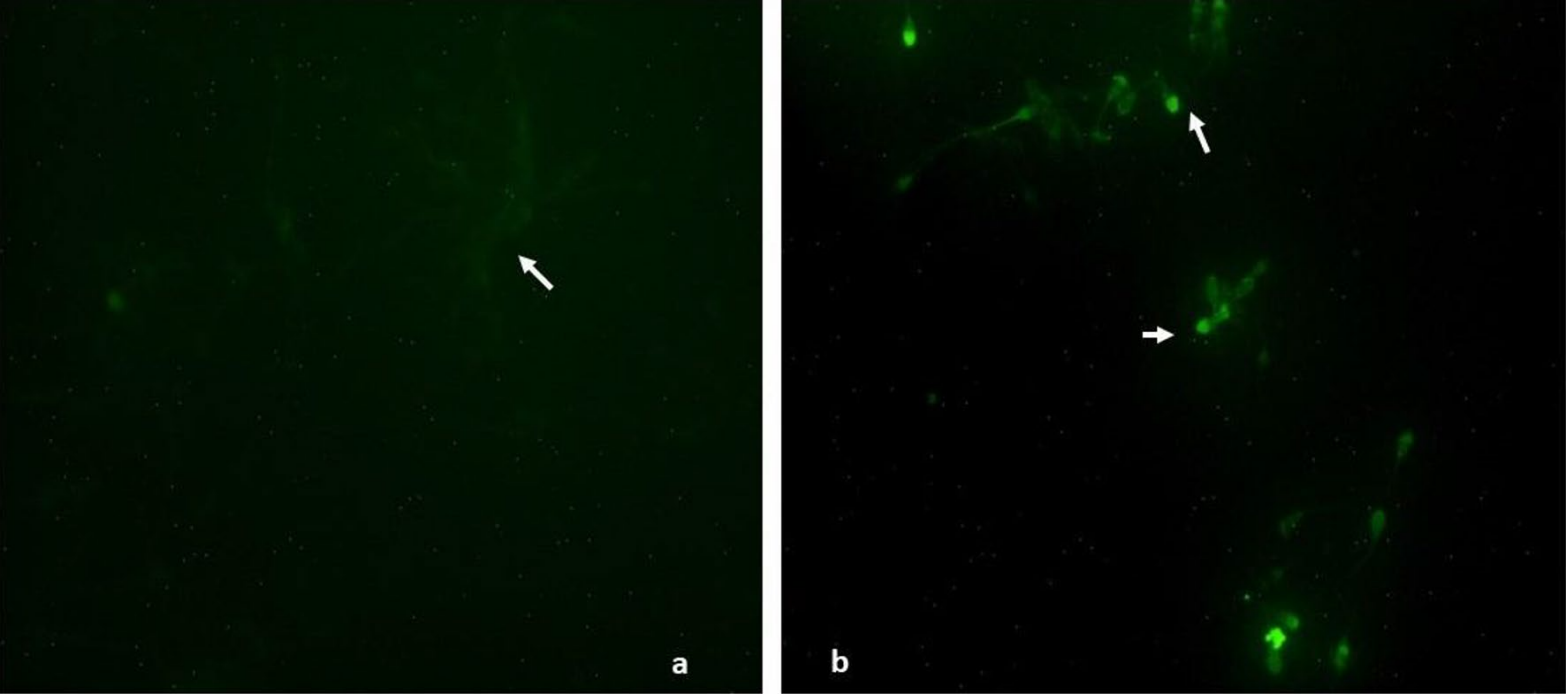
DNA damage in goats’ spermatozoa. (**a**) Showing no-fluorescence in spermatozoa (TUNEL-negative) in PBS control and 0.031, 1.25 and 0.25 µg/ml HgCl_2_ groups after 3 h. (**b**) Showing bright green-fluorescence in head of the spermatozoa (TUNEL-positive; arrow) of mercuric chloride (1.25 µg/ml) treated group after 3 h of exposure.

Data presented in Table 4 and shown in Fig. 3 revealed that mercury even at the lower concentration (0.031 µg/ml) did not induce any DNA damage even after 3 h of exposure. Similarly, compared to the PBS control, even highest used concentration of mercury (1.25 µg/ml) also failed in inflicting any significant DNA damage in sperm cells after 15 min (0.71%). However, compared to the control (0%), significant (p < 0.05) increase in number of sperm cells showing DNA damage (29.04%) was observed after exposure to 1.25 µg/ml HgCl_2_ for 3 h.

**Table 4:** Effect of in-vitro exposure of buck spermatozoa to different concentrations of mercuric chloride (0.031–1.25 µg/ml) on DNA damage in sperm cells at different time intervals.

Treatments	% spermatozoa showing DNA damage at different time intervals	
	15 min	3 h
PBS control	0.00 ± 0.00^Aa^	0.00 ± 0.00^Aa^
0.031 µg/ml mercuric chloride	0.00 ± 0.00^Aa^	0.00 ± 0.00^Aa^
0.125 µg/ml mercuric chloride	0.00 ± 0.00^Aa^	0.00 ± 0.00^Aa^
0.25 µg/ml mercuric chloride	0.00 ± 0.00^Aa^	2.03 ± 1.01^Aa^
1.25 µg/ml mercuric chloride	0.71 ± 0.33^Aa^	29.04 ± 1.06^Bb^

Data presented are mean ± SE of the semen samples of six bucks.

Different capital superscripts in the rows indicate significant (p < 0.05) differences between the different time intervals.

Different small superscripts in the columns indicate significant (p < 0.05) differences between control and different treatment groups.

### Scanning electron microscopy (SEM)

**Figure 4:**
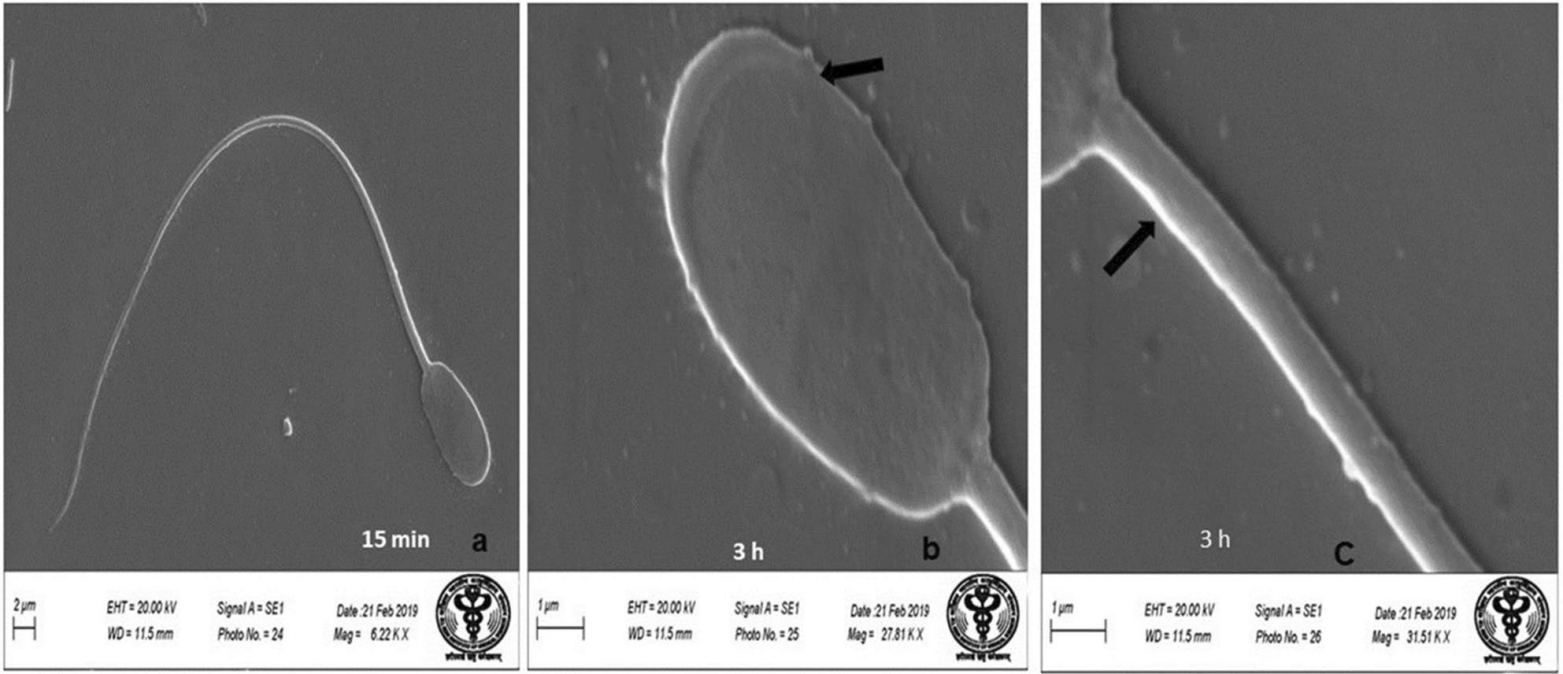
Buck spermatozoa of control group showing normal surface morphology. Scanning Electron Microscope images of buck spermatozoa of control group shows normal surface morphology (**a**), intact acrosome (**b**) and intact midpiece (**c**) after 15 and 3 h.

**Figure 5:**
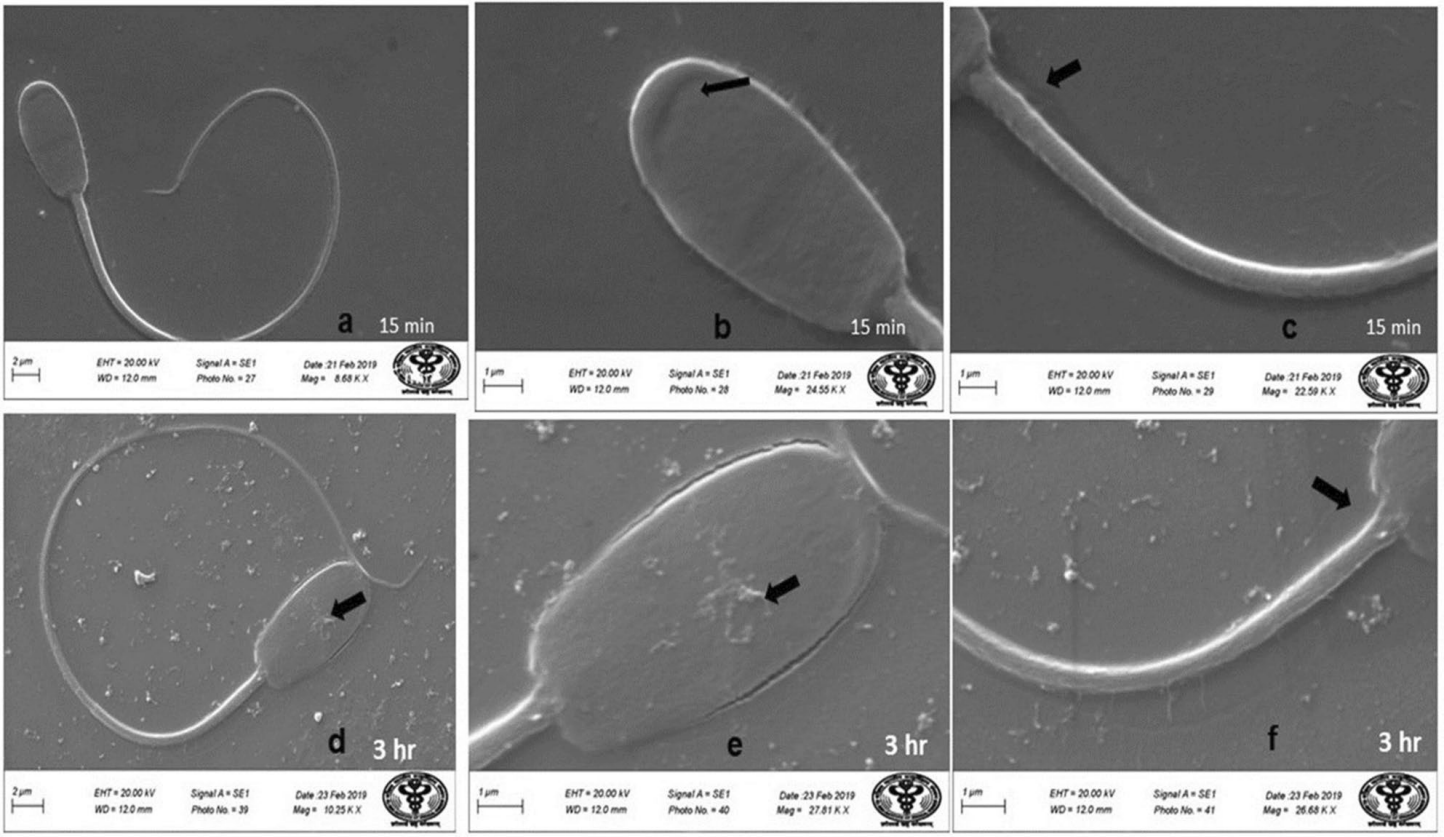
Scanning Electron Microscope images of goat spermatozoa in-vitro treated with Hg (0.031 µg/ml HgCl_2_) showing intact head and tail and normal surface morphology (**a**), intact acrosome (**b**) and intact midpiece (**c**) after 15 min exposure. While wrinkled plasma membrane on head region (**d**,**e**) and middle piece deformities and focal area of membrane rupture at middle piece were more obvious after 3 h exposure of mercury (**f**).

**Figure 6:**
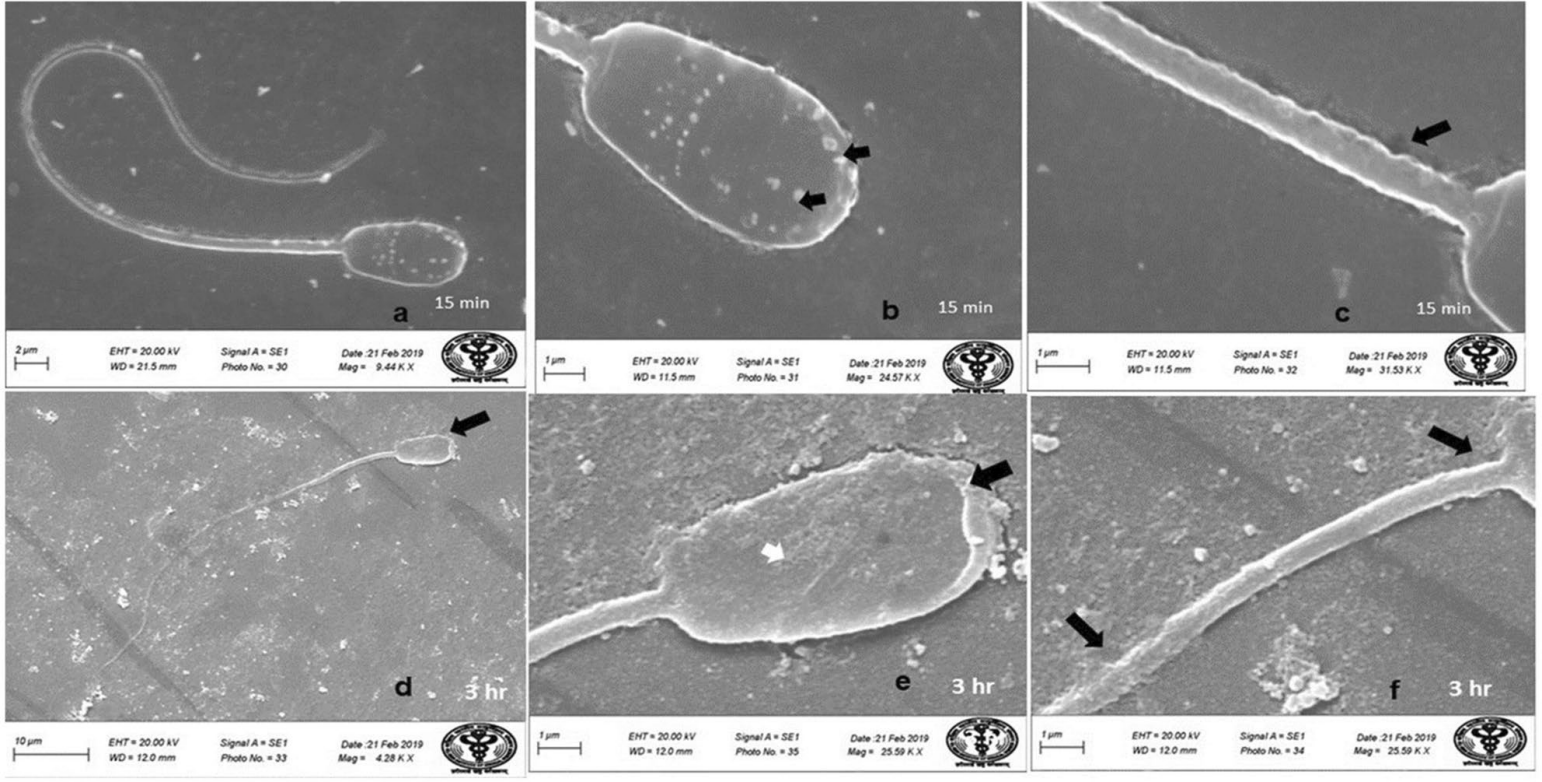
Scanning Electron Microscope images of goat spermatozoa in-vitro treated with Hg (1.25 µg/ml HgCl_2_) showing deformities in overall spermatozoa (**a**), membrane damage (**b**), wavy middle piece (**a**,**c**) after 15 min exposure. Swollen acrosome and wrinkled head plasma membrane (**d**,**e**), damaged tail and the neck having irregular plasma membrane (**f**) after 3 h exposure of 1.25 µg/ml HgCl_2_.

Evaluation of SEM images of the spermatozoa of control group revealed that spermatozoa had normal surface morphology (Fig. 4). Head of the spermatozoa was normal and oval in shape with intact acrosome (Fig. 4). Middle piece showed intact plasma membrane and there were no obvious deformities near the neck and tail joining regions (Fig. 4). Control group spermatozoa had over all homogenous plasma membrane. Following in-vitro exposure of sperm cells to lower concentration of HgCl_2_ (0.031 µg/ml) for 15 min, apparently no adverse effect was observed on the surface morphology of spermatozoa (Fig. 5a) and no obvious changes were observed in sperm head, acrosome and tail parts at this time point and it compared well with the spermatozoa of control group (Fig. 5b,c). When spermatozoa were exposed to the same concentration of mercuric chloride (0.031 µg/ml) for longer period of time i.e. 3 h, several defects were noticed (Fig. 5d) as revealed by deformities in the head and middle piece regions of sperm cells at this time-point (Fig. 5e,f). In some of the spermatozoa, wrinkled plasma membrane in the head region (Fig. 5e) and focal areas of membrane rupture at the middle piece were observed (Fig. 5f). However, exposure to higher concentration of HgCl_2_ (1.25 µg/ml) resulted in deformities in spermatozoa head plasma membrane and middle piece even after 15 min of exposure (Fig. 6a). The SEM analysis also showed that at this time point, 1.25 µg/ml HgCl_2_ hampered the head plasma membrane integrity and there were focal points of membrane damage (Fig. 6b). Middle piece of sperm cells harbouring mitochondria also appeared wavy depicting the harmful effects of higher concentration of mercury on sperm cells (Fig. 6a,c). Further, the morphological defects in sperm cells were more pronounced after 3 h of exposure to 1.25 µg/ml of HgCl_2_ (Fig. 6d) as the head plasma membrane was found to be wrinkled and acrosome was swollen (Fig. 6e). Tail part of the sperm cell also appeared damaged and the neck appeared to have irregular plasma membrane (Fig. 6f).

### Transmission electron microscopy (TEM)

**Figure 7:**
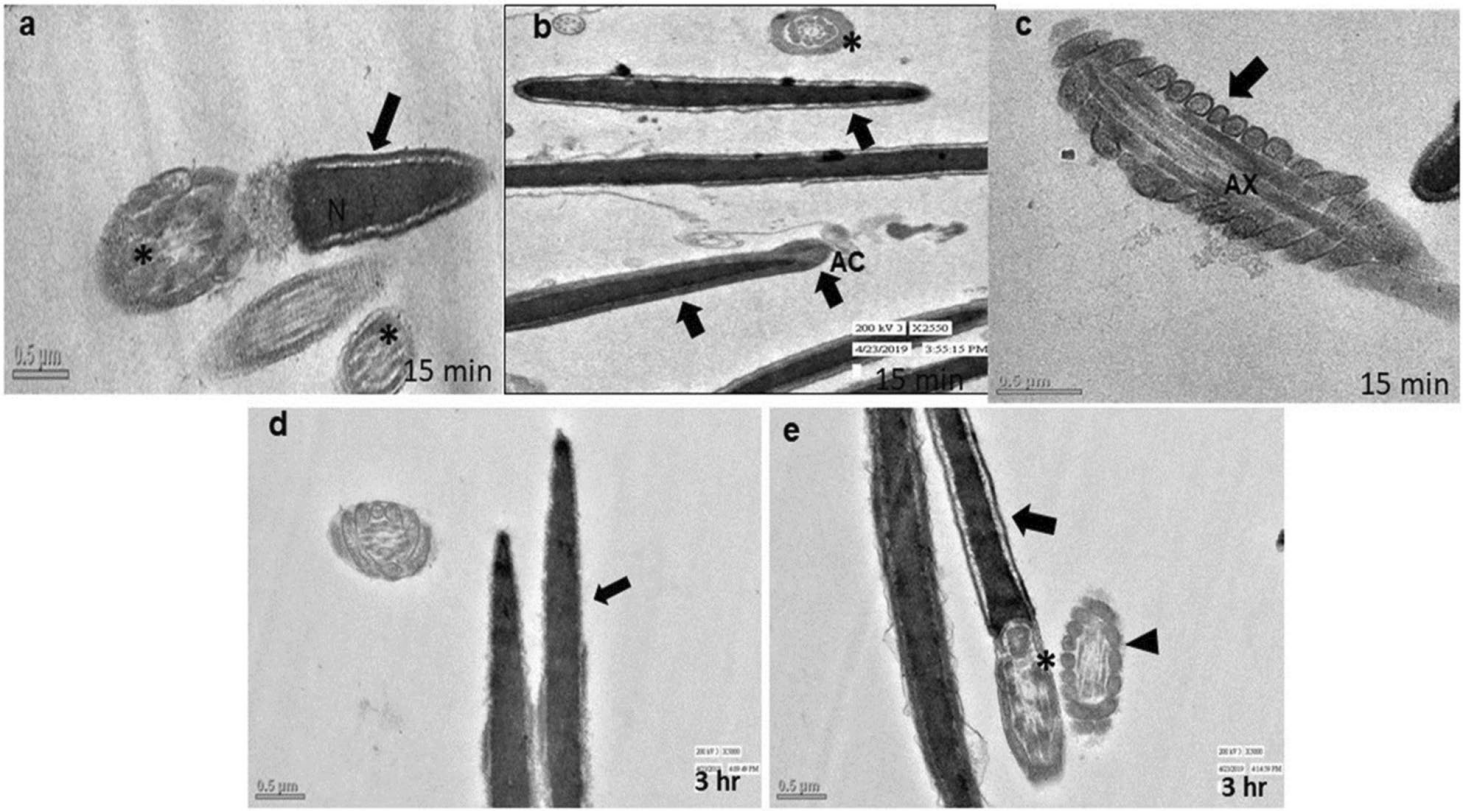
Transmission electron micrographs of the goat spermatozoa of control group after 15 min and 3 h showing intact ultrastructures. (**a**) Longitudinal section of the sperm nucleus; head (arrow; (**a**)); axonemal components (star; (**a**,**b**)), (**b**) intact sperm head (arrow) and intact acrosome (AC; (**b**)), organized mitochondria (arrow; (**c**)), sperm head (arrow; (**d**,**e**), axonemal components (star; (**e**)); organized mitochondria (triangle; (**e**)), intact plasma membrane (arrow; (**e**)).

**Figure 8:**
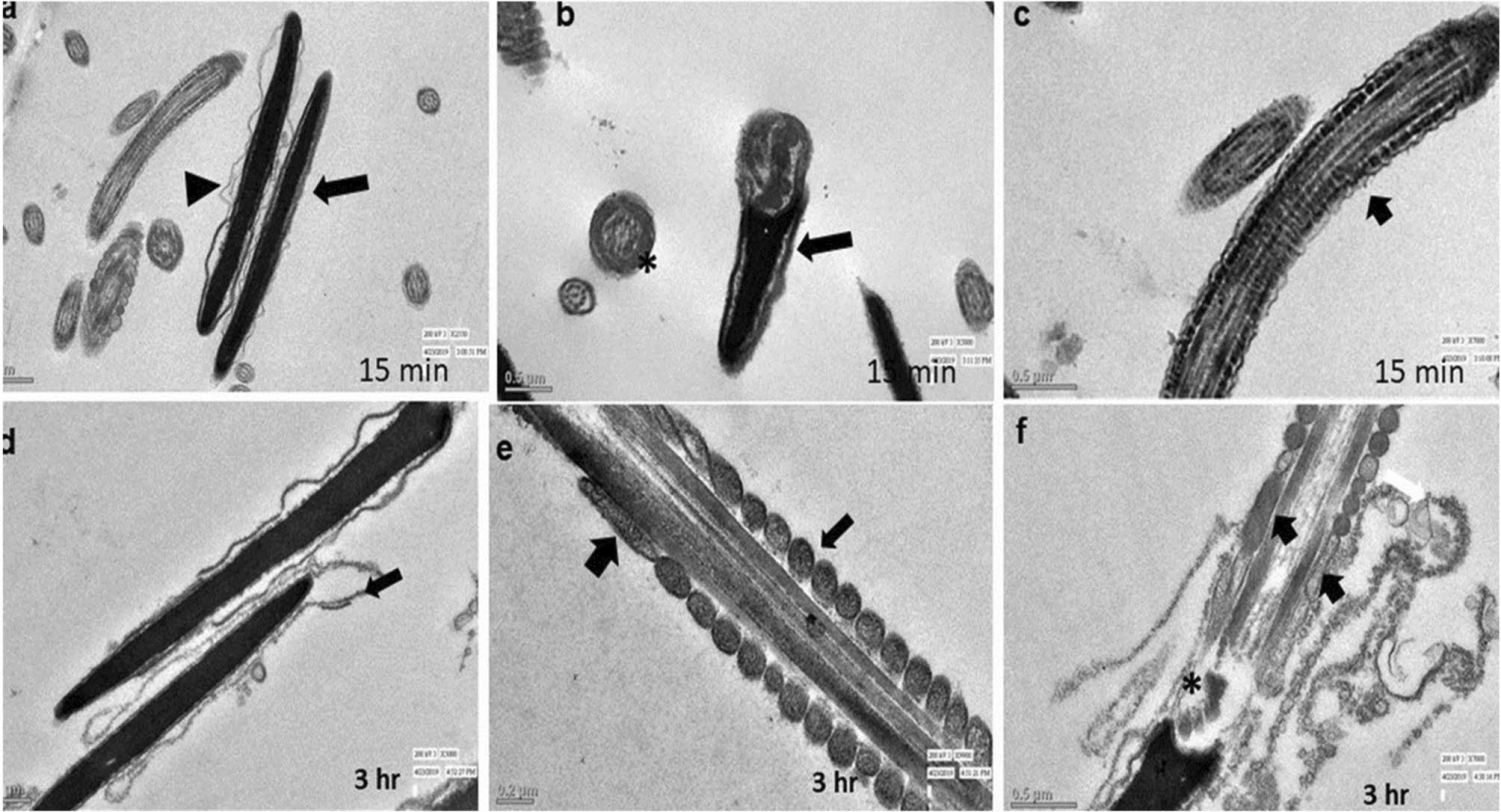
Transmission electron micrographs of goat spermatozoa exposed to 0.031 µg/ml HgCl_2_ showing ultra-structural defects. (**a**) sperm head (arrow); damage membrane (triangle), (**b**) sperm head (arrow); axonemal components (star), (**c**) outer dense fibers (arrow), (**d**) reacted acrosome (arrow), (**e**) mitochondria (arrow); collapsed mitochondria (arrow), (**f**) disorganized outer dense fiber(star); collapsed mitochondria (arrow) after 15 min and 3 h.

**Figure 9:**
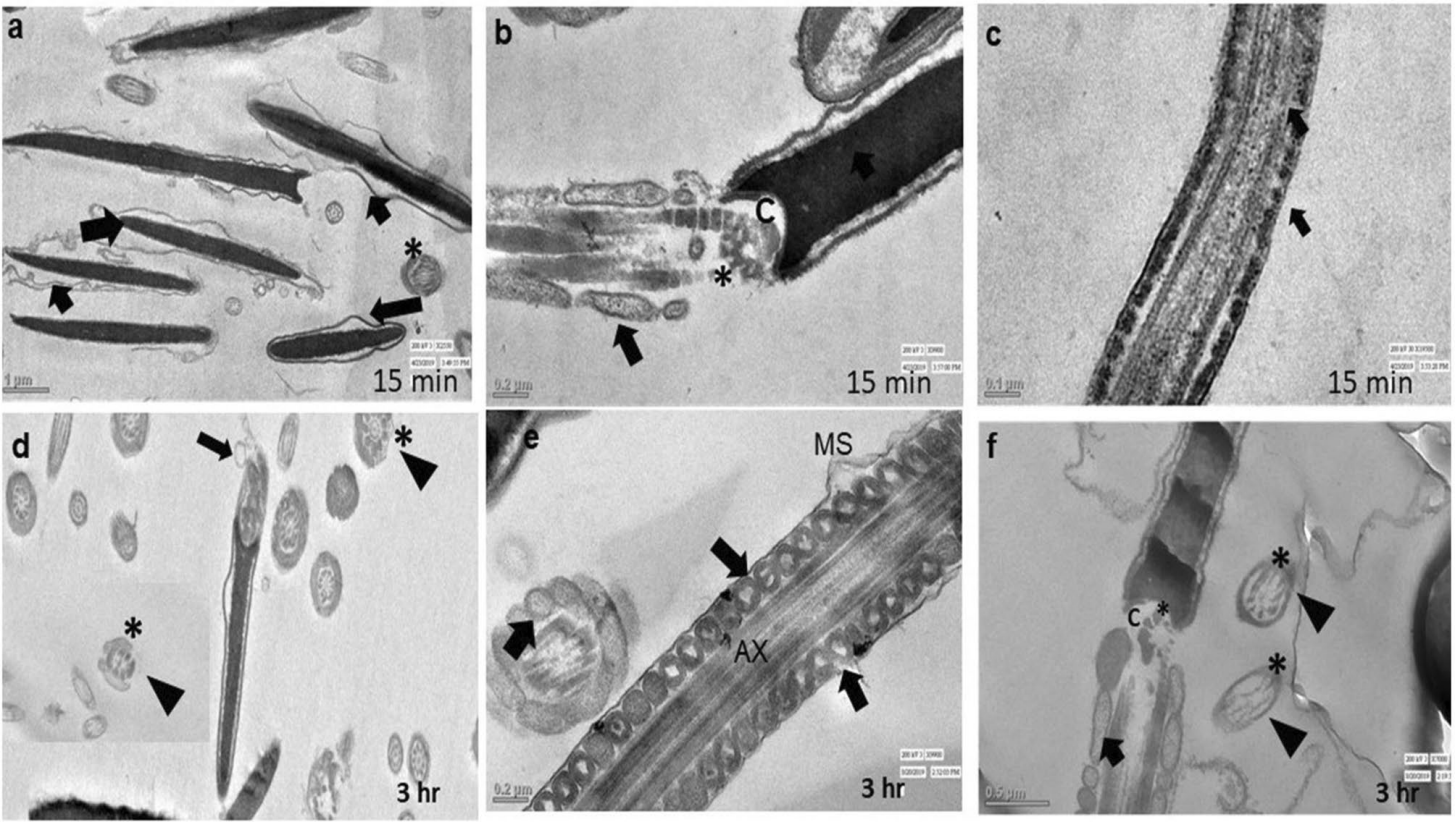
Transmission electron microscopic images of goat spermatozoa exposed to 1.25 µg/ml HgCl_2_ showing ultra-structural defects. (**a**) swollen sperm head membrane (arrow), (**b**) collapsed mitochondria (arrow); bent neck (star); disrupted connection between nucleus and midpiece by centriole (**c**,**b**,**f**), (**c**) outer dense fibers (arrow), (**d**) damaged head membrane (arrow); damaged axoneme membrane (triangle; (**d**,**f**)), (**e**) mitochondria (arrow); axoneme (AX; arrow); damaged mitochondrial sheath (MS; (**e**)),collapsed mitochondria (arrow; (**f**)); disorganized exoneme (star; (**f**)) after 15 and 3 h.

Evaluation of TEM images of the spermatozoa of control group did not show any notable abnormalities in sperm cells after 15 min or 3 h. Spermatozoa of the control group (15 min) showed intact plasma membrane and well-organized axonemal components (Fig. 7a,b). The longitudinal section of spermatozoa tail showed normal axoneme arrangements and intact mitochondrial ultrastructure at the same time point (Fig. 7c). Even after 3 h, it showed intact membrane, acrosome, axonemal components (*) and well-organized mitochondria (Fig. 7a,b), intact head (Fig. 7d), mitochondria and axonemal arrangements (Fig. 7e). Spermatozoa of 0.031 µg/ml HgCl_2_ treated group after 15 min of exposure showed almost normal ultra-structures (Fig. 8a–c) and the TEM observations were in accordance with the SEM observations. However, few spermatozoa at this time point showed irregular head plasma membrane (Fig. 8a). But no notable changes were observed in the longitudinal sections of sperm tail at this time point (Fig. 8c). But following exposure to HgCl_2_ (0.031 µg/ml) for 3 h, certain adverse effects were observed on ultra-structures of the buck spermatozoa as revealed by ruptured plasma membrane and premature acrosome reaction in longitudinal sections of spermatozoa (Fig. 8d). The longitudinal sections of the middle piece revealed disorganized and swollen mitochondria having collapsed cristae in the middle piece of some of the spermatozoa (Fig. 8e,f). Disorganized outer dense fibres were also observed at this time point in few of the spermatozoa (Fig. 8f). However, higher concentration (1.25 µg/ml) of HgCl_2_ damaged spermatozoa head and tail integrity even after 15 min of exposure (Fig. 9a–c). Some of the spermatozoa showed disorganized axonemal components and bent neck (Fig. 9b). The distorted outer dense fibres in tail part were also observed in few of the spermatozoa (Fig. 9c). Compared to the ultrastructural damages observed in sperm cells after exposure to 1.25 µg/ml HgCl_2_ for 15 min, more severe and adverse ultra-structural defects were observed after 3 h of exposure (Fig. 9d–f) and these were in agreement with the SEM observations. Longitudinal sections of the spermatozoa head showed fragmented plasma membrane and focal areas of lysis (Fig. 9d). The middle piece region had swollen mitochondria with collapsed cristae (Fig. 9e,f). Additionally, disorganized axoneme and disorganised outer dense fibres were also noticed at this time point in some of the spermatozoa (Fig. 9f). Based on the observed lesions observed in TEM images, it is apparent that the toxic effect of mercury on spermatozoa was both time- and concentration-dependent and mercury damaged almost all parts of the sperm cells including mitochondria.

### Apoptosis and necrosis

**Figure 10:**
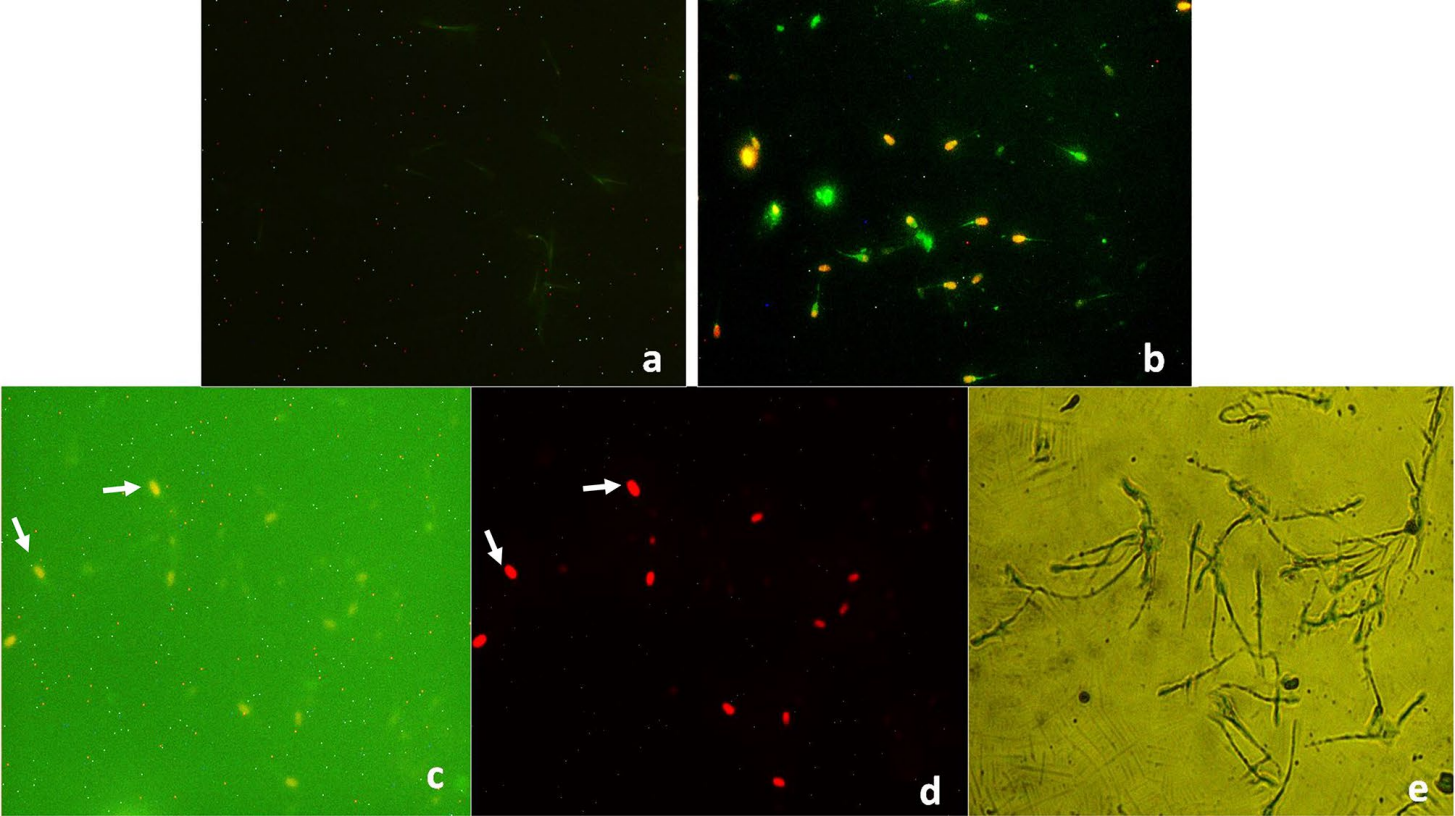
Detection of apoptosis and necrosis using Annexin˗V staining in goat spermatozoa. (**a**) Spermatozoa with no sign of phosphatidylserine translocation [Annexin-V-FITC (negative) and PI (negative)] in control group. (**b**) Annexin-V-FITC (green) and PI (red) positive spermatozoa in positive control (Camptothecin 10 µM). (**c**) Annexin-V-FITC (green; negative) and PI (positive; red) spermatozoa in mercuric chloride treatment groups after 3 h (0.031–1.25 µg/ml) in green filter. (**d**) Annexin-V-FITC (green; negative) and PI (positive; red) spermatozoa in mercuric chloride treatment groups after 3 h (0.031–1.25 µg/ml) in red filter. (**e**) DIC image of (**c**,**d**); mercury treated groups.

No apoptosis was observed in the spermatozoa of PBS control and mercury-treated groups after 15 min of exposure. After 3 h, compared to just 0.67% apoptotic sperm cells in semen samples of the control group, 41.67% apoptotic spermatozoa were observed in the positive control group (Camptothecin) as evident from the data summarized in Table 5 and shown in Fig. 10. But compared to the untreated control or positive control (Camptothecin) groups, no apoptosis was observed in sperm cells of any of the mercury-treated groups even after 3 h of incubation. Rather, large number of sperm cells in mercury-treated groups were found to be necrotic (clearly visible under red filter; Fig. 10d) and their counts were 18.31, 23.45, 32.13 and 39% in 0.031, 0.125, 0.25 and 1.25 µg/ml HgCl_2_-treated groups, respectively after 3 h of exposure (Table 5).

**Table 5:** Effect of in-vitro exposure of buck-spermatozoa to different concentrations of mercuric chloride (0.031–1.25 µg/ml) on early apoptosis and necrosis at different time intervals.

Treatments	% Apoptotic and necrotic spermatozoa at different time intervals			
	15 min		3 h	
	Necrosis	Apoptosis	Necrosis	Apoptosis
Positive control*	5.00 ± 0.58	29.67 ± 0.33	8.33 ± 0.88	41.67 ± 2.40
PBS Control	6.00 ± 1.53^D^	0.00 ± 0.00	11.00 ± 2.65^D^	0.67 ± 0.00
0.031 µg/ml Mercuric chloride	10.00 ± 1.15^C^	0.00 ± 0.00	18.31 ± 2.85^CD^	0.00 ± 0.00
0.125 µg/ml Mercuric chloride	14.67 ± 0.33^B^	0.00 ± 0.00	23.45 ± 3.38^BC^	0.00 ± 0.00
0.25 µg/ml Mercuric chloride	18.68 ± 0.33^AB^	0.00 ± 0.00	32.13 ± 3.38^AB^	0.00 ± 0.00
1.25 µg/ml Mercuric chloride	22.00 ± 0.58^A^	0.00 ± 0.00	39.00 ± 2.65^A^	0.00 ± 0.00
p-value	0.000	0.0	0.000	0.0

Data presented are mean ± SE of the semen samples of six bucks.

Different capital superscripts in the columns indicate significant (p < 0.05) differences between the different treatment groups.

*Camptothecin (10 µM) treated buck semen sample.

### Correlation between DNA damage, apoptosis and necrosis

Data presented in Table 6 although indicated negative correlation between apoptosis and necrosis after 15 min of exposure to mercury, but it was statistically insignificant, Likewise, apoptosis was negatively corelated with necrosis in HgCl_2_-treated groups after 3 h of exposure as well. But DNA damage was significantly corelated with necrosis while negatively correlated with apoptosis, although it was statistically insignificant.

**Table 6:** Correlation between necrosis, apoptosis and DNA damage of different concentrations of mercuric chloride (0.031–1.25 µg/ml) at different time intervals (*p ≤ 0.05).

15 min	Necrosis	Apoptosis	DNA damage
Necrosis	1		
Apoptosis	− 0.42	1	
DNA damage	0.53*	− 0.149	1

3 h	Necrosis	Apoptosis	DNA damage
Necrosis	1		
Apoptosis	− 0.86*	1	
DNA damage	0.75*	− 0.27	1

### Comparative expression of apoptotic and anti-apoptotic genes

**Figure 11:**
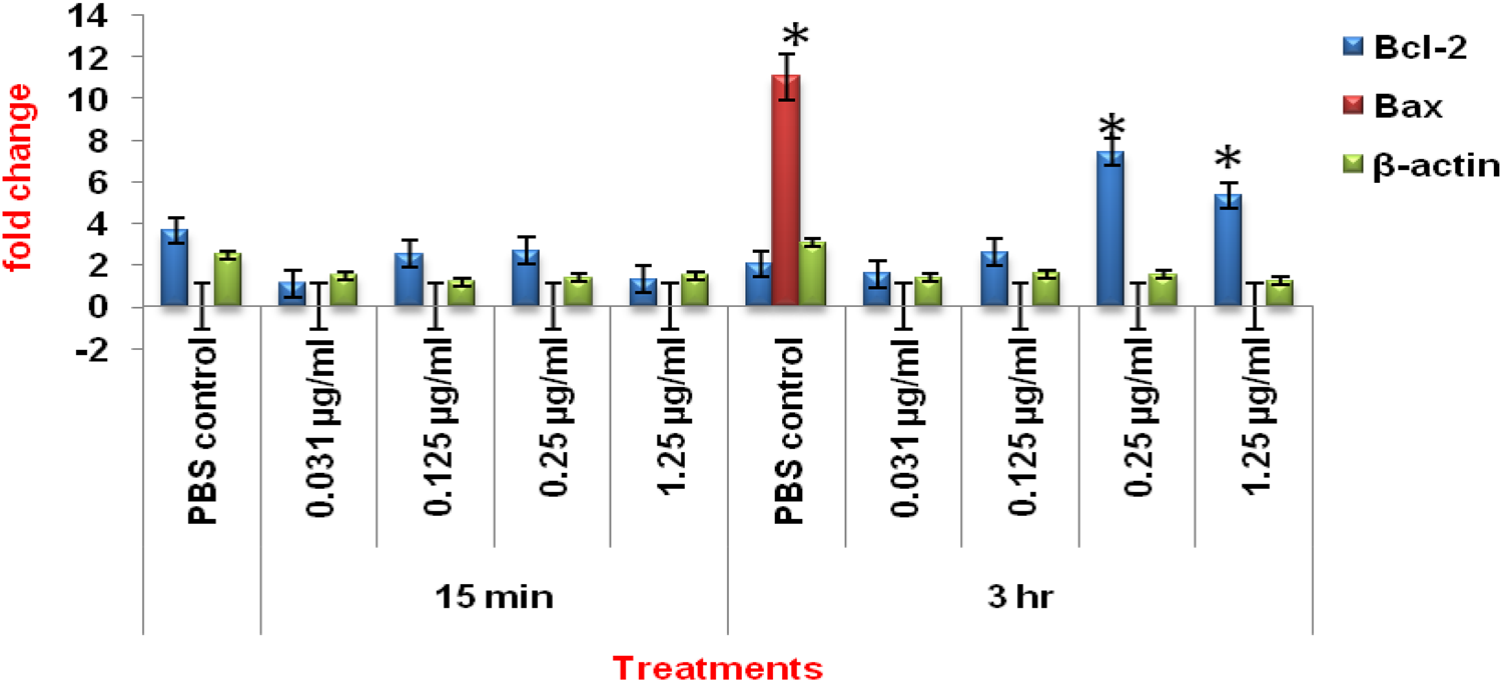
Relative mRNA expression of the apoptotic (*Bax*), anti-apoptotic genes (*Bcl*-2) and housekeeping gene (β-*actin*) in the spermatozoa following treatment of semen samples with different concentrations of mercuric chloride at different time intervals. Vertical bars represent the mean ± SE. Asterisk (*) on bars represent the significant differences between the PBS control and different treatment groups at different time intervals.

Data presented in Fig. 11 revealed that compared to the control group, no significant (p > 0.01) change was observed in the relative mRNA expression of Bcl-2 gene in semen samples exposed to different concentrations of HgCl_2_ (0.031, 0.125, 0.25 and 1.25 µg/ml) for 15 min. But after 3 h, compared to the control, there was significant (p < 0.01) increase in the relative mRNA expression of Bcl-2 in 0.25 and 1.25 µg/ml mercuric chloride-treated groups. Compared to β-actin and Bcl-2 genes, relative mRNA expression of Bax gene was not altered either in the control or in HgCl_2_ treated groups after 15 min of exposure. But after 3 h, although significant (p < 0.01) increase in the relative expression of Bax gene was observed in the control group but there was no change in the relative mRNA expression of Bax gene in any of the mercury-treated groups even after 3 h. Rather significant (p < 0.01) increase in the relative mRNA expression of Bcl-2 gene was observed in HgCl_2_ (0.25 and 1.25 µg/ml) treated groups**.**

## Discussion

According to U.S. Geological Survey and Harvard University^27^, mercury levels in the northern Pacific Ocean have increased by about 30 per cent over the past 20 years and it is expected to rise further by 50 per cent by 2050 with increase in industrial mercury emissions. Mercury levels in air are in the range 2–10 ng/m^3^^28^. Although naturally occurring levels of mercury in groundwater and surface water are less than 0.5 µg/l, but local mineral deposits may result in higher levels of mercury in groundwater^28^. The permissible limit for mercury in drinking water is 0.01 ppm^28^. Average daily intake of mercury from food is in the range 2–20 µg, but may be much higher in regions where ambient waters are more contaminated with mercury and where fish constitute major proportion of human’s diet^29^. Fish mostly contain varying levels of mercury like 0.031 ppm in pollock, 0.126 ppm in tuna, 0.144 ppm in fresh/frozen skipjack, 0.150 ppm in freshwater perch, and 1.123 ppm in tilefish in Gulf of Mexico^29,30^. United Nations Environment Programme’s (UNEP) Chemicals Working Group has pointed out that mercury contamination in India is reaching at alarming levels largely due to the discharge of mercury-bearing industrial effluents ranging from 0.058 to 0.268 mg/l^31^; and this is several times more than the prescribed Indian and WHO standards for drinking water and for industrial effluents. Thus, all the above-mentioned reports suggest that human beings and animals in India and other countries are being exposed to higher concentrations of mercury.

In our recent publication, we have reported mercury-induced significant increase in ROS and MDA, significant decrease in TAC and SOD activity, significant decrease in spontaneous acrosome reaction, and inhibited capacitation in sperm cells despite increase in cAMP and intracellular Ca^2+^ levels even at 0.031 µg/ml compared to the control group after 15 min exposure. Based on these findings, we proposed that oxidative stress causes alterations in sperm functions^13^ possibly by affecting sperm membrane as well as sub-cellular structures. To substantiate our proposed mechanism of mercury-induced alterations in functional dynamics of spermatozoa, present study was undertaken to understand how cell death takes place and what changes take place in sperms at molecular and ultra-microscopic levels, especially at organelle level, that affect the motility and kinematic patterns in an endeavour to give a fillip to our understanding about mercury-induced cell death and alterations in functional dynamics of spermatozoa.

Mercury has been associated with compromised sperm functions and reproductive failures^32^. Men having mean mercury level of 8.1 ng/ml have been reported to be infertile compared to those having 6.3 ng/ml in control^33^. Infertile males have been reported to have higher level of mercury (0.048 µg/l) in their seminal plasma compared to the fertile males having 0.032 µg/l in seminal plasma^34^. Decreased sperm motility, sperm swimming speed, and increased abnormal sperm tail morphology have been reported in monkeys having blood mercury levels around 2000 ng/ml^35^. Rats having mean blood mercury levels of 30.8 ng/ml^36^, and 94.3 and 176.5 ng/ml^37^ have also been reported to have significant variations in testosterone levels and adverse effects on male reproduction. In all these studies, spermatozoa were exposed to comparatively higher concentrations of mercury than used by us in the present study. Several reports on toxic effects of mercury on semen quality and functional dynamics of sperm cells in humans and experimental animal are also available^8–10,12,33,38^. But the specific target site(s) for action of mercury and its mechanistic pathway(s) are yet to be precisely delineated.

Computer assisted semen analyser CASA provides quantitative data of sperms motility (rapid-, slow- and non-progressive) and specific kinematic measures. Our data provides quantitative evidence in favour of mercury-induced reduction in proportion of the motile sperms and their straight-line motion. Major effects of mercury on motility parameters (rapid, slow and non-progressive) and kinematic motion parameters (VCL, VSL, VAP and LIN) were highly correlated. Total motility, rapid and non-progressive were associated with the exposure level of mercuric chloride (0.31 to 1.25 µg/ml) and time of exposure. The VCL, VSL, VAP and LIN of sperms are bioindicators of the fertilizing ability and have been correlated with in-vivo fertilization rates in humans^39^. After 3 h, we observed dose-dependent decline (∼ 31% shorter) in VSL of the sperms of 1.25 µg/ml HgCl_2_ exposed group as the spermatozoa travelled at the rate of 84.25 μm/s compared to 122.17 μm/s in the control group. The VAP and VCL data also suggest that sperm of 1.25 µg/ml HgCl_2_ treated group moved ∼ 24% slowly along their average path and point to point path of travel compared to control (Table 3). The concentration-dependent significant decline in ALH and BCF suggest that mercury alters the frequency at which sperms cross the average path (∼ 25 times per second) and maximum amplitude of deviation around that average path (∼ 3.8 μm) in dose- and time-dependent manner. Taken together, with mercury concentration-dependent increase in LIN, STR and WOB trend, our findings suggest that sperms following exposure to higher concentration of mercury follow more linear path (i.e. less curved) and possess less beat cross frequency than the sperms of control group.

The ALH measures the vigour of flagellar beating in conjunction with the frequency of cell rotation^40^, and both these are associated with the ability of sperm to penetrate cervical mucus and fuse with oocytes. Therefore, ALH is often used to assess the outcome of in vitro fertilization programmes^41^. Higher concentration (1.25 µg/ml) of HgCl_2_ drastically reduced the sperm population that is likely to reach the oocyte as a consequence of the reduced percentage of rapid motile sperms and reduced BCF and maxALH motion kinetic in the remaining motile sperm cells. Thus, our CASA data evidently suggests that mercury directly diminishes the ability of sperms to penetrate and fuse with oocytes. Our observations of the kinematic patterns also validate our earlier finding^13^ in which we did not observe any capacitation in sperms following exposure to mercuric chloride at 1.25 µg/ml for 15 min and 3 h.

Mercury (217.216 ppm; 800 μΜ) has been reported to significantly (p < 0.001) increase (12.0% compared to 2% in control) percentage of DNA breaks in human sperms^42^. Positive correlation between sperm DNA fragmentation and levels of ROS has been reported in testicular tissue of mice^43^ and semen of humans^44,45^. TUNEL assay in the present study revealed that mercuric chloride at lower concentration (0.031 µg/ml) did not induce any DNA damage in sperm cells even after 3 h of exposure. But DNA damage was observed in 2.03 ± 1.01 and 29.04 ± 1.06% sperm cells following exposure to 0.25 µg/ml and 1.25 µg/ml concentrations of mercuric chloride, respectively, for 3 h. Our findings on DNA damage at higher concentrations are in agreement with the effect of 150, 350 and 550 μM HgCl_2_ induced DNA breaks in bull sperm nuclei where interestingly 92% of the DNA breaks were double-stranded^8^. Hayati et al.^46^ also reported mercury-induced increase in DNA fragmentation in fish sperms after in-vitro exposure to different concentrations of HgCl_2_ (0.5, 1, 2.5 and 5 ppm) for 5 s. DNA damage observed in sperm cells in the present study can be attributed to mercury-induced significant increase in oxidative stress biomarkers in goat semen^13^. But possibly such a low concentration of inorganic mercury (0.031 µg/ml) was not sufficient to induce DNA damage in goat spermatozoa despite significant oxidative stress and viability losses in goat spermatozoa^13^. It seems interesting and but difficult to put forth any plausible explanation for the same.

Arabi et al.^1^ reported 64% viability losses in bull sperm cells after exposure to mercuric chloride (13.576 µg/ml) for 2 h. Similar viability loss in of goat spermatozoa was also observed and it was found to be reduced by 50% after 3 h of exposure but at comparatively much lower concentration (1.25 µg/ml). Thus, goat sperm cells seem to be much more sensitive to mercury-induced cellular insult than bull spermatozoa. Higher vulnerability of goat sperms vis-à-vis bull sperms at the moment can be only attributed to species-difference, however, to unravel the precise possible reason for such a variability in response to mercury requires comparative studies on sperm cells of both these species.

Scanning and transmission electron microscopy images of mercury treated spermatozoa revealed loss of plasma membrane integrity along with acrosomal membrane damage, damage of the head region, damaged outer dense mitochondrial sheath along with collapsed cristae, disorganized and swollen mitochondria, reacted acrosome, disorganized axonemal components with altered implantation fossa. These defects in mitochondria seem to be responsible for reduced mitochondrial transmembrane potential and motility of the spermatozoa. Abnormalities in head and neck region may be responsible for detachment of the head and tail leading to instant death of some spermatozoa. Our observations on ultra-structural alterations in goat spermatozoa are almost similar to those reported in rabbit sperms after treatment with higher than 1 µM mercury, arsenic, cadmium, and platinum as in rabbit sperms also damage to sperm head membranes and acrosome breakage with formation of various sized micro-vesicles was observed^47^.

In the present study, we observed that mercury did not induce apoptosis even at the highest used concentration (1.25 µg/ml); rather it induced necrosis even at the lowest used concentration (0.031 µg/ml). Generally, little percentage of labelling with annexin is observed in every basal sample but in the present study, compared to the control, none of the spermatozoa from mercury-treated groups showed any exteriorization of phosphatidyl serine. This also substantiates that mercury did not induce early apoptosis like changes in mercury-treated sperm cells. Rather, mercury resulted in instant cell death as substantiated by propidium iodide positive (PI^+^) cells data during Annexin V assay as the sperm cells were mainly labelled with PI and very less/no with annexin which indicates that sperm membrane ruptures as indicated by electron microscope images might be responsible for necrosis. Rather mercury appears to be protective against P-serine externalization, however, to substantiate the protective role of mercury against P-serine externalization, further studies are required specifically taking spermatozoa of some other species as well from comparative perspective.

Theory of apoptosis-induced sperm DNA damage has been challenged by several researchers^48–50^. Apoptosis-independent DNA damage observed in the present study is in agreement with the similar observations of Pereira et al.^51^ who have reported mercury induced DNA damage in blood cells by a nonapoptotic mechanism in wild and caged fish. On U-937 cells also, mercury was found to damage DNA, but apoptosis was not involved. Similarly, Muratori et al.^48^ have also reported that sperm DNA fragmentation did not correspond to the apoptosis-like phenomenon and the impaired motility of sperms in human ejaculate was associated with ultrastructural damages. Sakkas et al.^49^ also observed that although DNA damage was initiated in some of the spermatozoa by mercury but subsequently this escaped apoptosis, and they termed it as “abortive apoptosis”. Lack of mercury-induced apoptosis in goat spermatozoa is also substantiated by our real time quantification data of the expression of pre-apoptotic (Bax) and anti-apoptotic (Bcl-2) genes where the former gene was expressed only in sperm samples of the control group after 3 h but not at all in the mercury-treated groups; thus implying routine and time-dependent apoptosis in sperm cells of control group while inhibition of Bax gene and significant concentration- and time-dependent increase in expression of Bcl-2 gene in mercury-treated groups. Thus, our findings are evidently indicative of the anti-apoptotic effect of mercury.

Mammalian Bcl-2 and related anti-apoptotic family member (Bcl-x_L_, Bcl-w, A1, Mcl-1, Boo/DIVA/Bcl-2, L-10, Bcl-B) proteins are localized on the cytoplasmic aspect of nuclear envelope, endoplasmic reticulum and outer mitochondrial membrane^52^. The fate of a cell depends on the balance of Bcl-2 family protein levels through a dynamic process by which cellular signals modulate the expression of pro- or anti-apoptotic proteins to tip the equilibrium towards survival or death^53,54^. When the balance favours death, the oligomerization of pro-apoptotic effector proteins on outer mitochondrial membrane leads to mitochondrial outer membrane permeabilization and release of cytochrome-*c*. This triggers activation of the cascade of caspases which cleave downstream substrates and ultimately cell death^53^. Tissue homoeostasis in eukaryotes requires multiple level regulation in tissue specific manner for fine-tuning of the apoptotic pathway. However, functional dynamics of Bax and Bcl-2 family members makes it extremely difficult to establish the physiological relevance of different modes of regulation reported either in cultured cell lines or in animal models^53,54^.

Our Bcl-2 gene expression data is in agreement with the observation of up-regulation of Bcl-2 mRNA expression in rat kidney cells^55^ and in neuronal and glial cells^56^ following exposure to mercury. Mercury (62.7 to 81.1 µM)-induced macrophage death has been reported through both- apoptosis and necrosis pathways^15^. But contrary to the observation of Kim and Raghubir^15^, loss of viability and alterations in motility and motion-kinematics in mercury-treated goats sperm cells was due to damaged ultra-structures in spermatozoa but this process is independent of apoptosis pathway.

Sperm motility is related to mitochondrial activity within the sperm midpiece^57^. In our earlier study, almost 40% goat sperm cells showed low mitochondrial transmembrane potential (MTP) following exposure to 1.25 µg/ml HgCl_2_^13^ and reduction in MTP correlated very well with the resultant reduction in sperm motility. Mercury has high affinity to bind with the sulfhydryl groups (–SH) of tubulins, the main component of sperm axonemal microtubules, and disrupts interactions between axonemal microtubular proteins and dyne in motors which are essential for sperm flagellar motility^11,35,58^. Therefore, reduction in sperm motility in mercury-treated sperms in the present study may be attributed to disorganized and collapsed cristae in mitochondria, release of cytochrome-*c* release due to permeabilization of mitochondrial membrane or inhibition of mitochondrial enzymes and uncoupling of oxidative phosphorylation^59,60^. However, to precisely understand the mechanism of mercury-induced sperm cell death by necrosis along with inhibition of Bax and upregulation of expression of Bcl-2, further studies are warranted.

**Figure 12:**
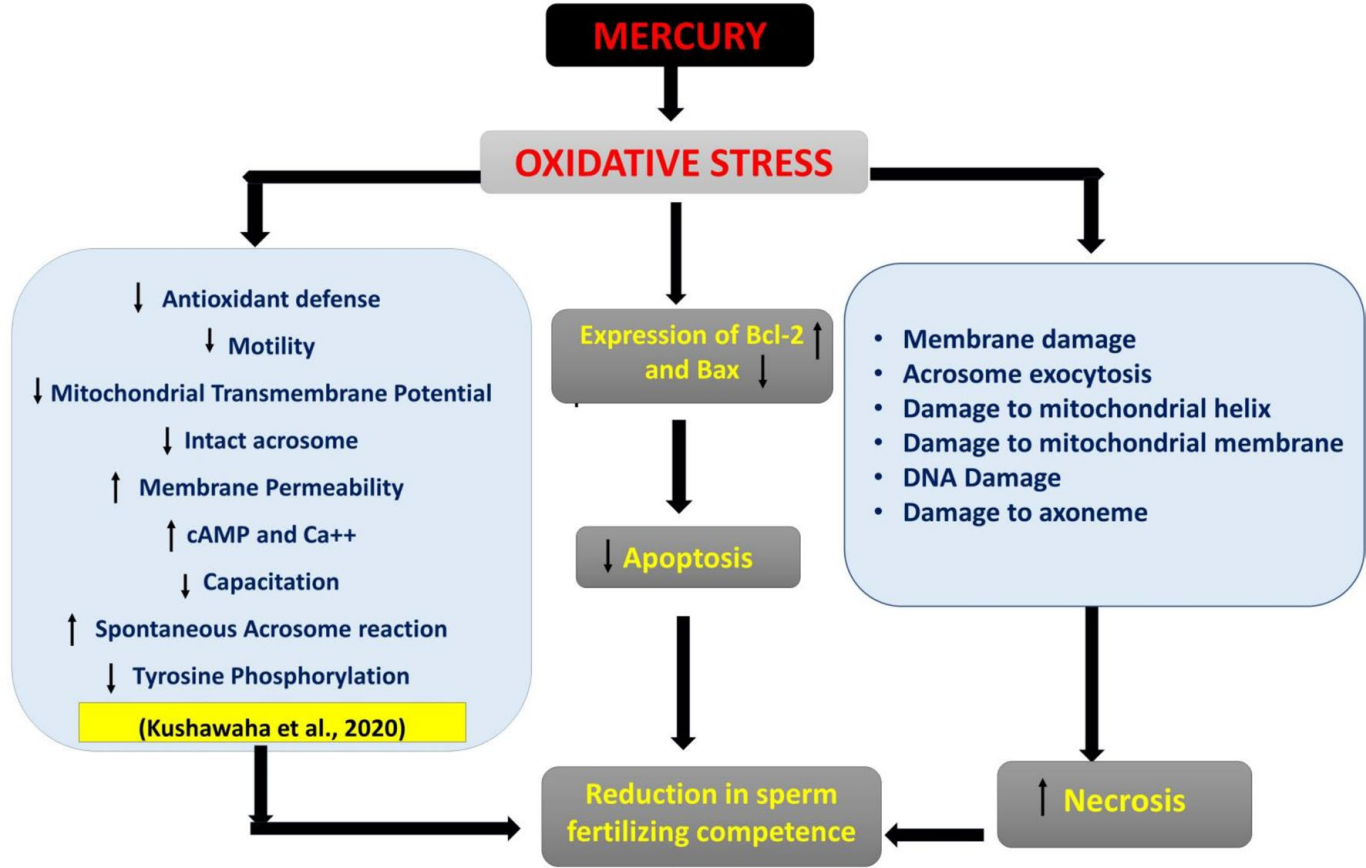
Proposed mechanistic pathways of mercury-induced toxicity to spermatozoa. Mercury-induced necrosis and death of spermatozoa seems to involve two independent pathways; mercury results in increase in levels of ROS and MDA and reduces total antioxidant capacity (TAC) and superoxide dismutase (SOD) activity and these ultimately result in decreased membrane intactness and cell death. Oxidative stress also results in lowered mitochondrial transmembrane potential (MTP) that may lead to increase in cAMP level and increased intracellular Ca^++^ release from spermatozoa that increase spontaneous acrosome reactions (AR) and reduction of capacitation which ultimately lead to reduction of sperm fertilizing ability. Altogether, mercury leads ROS-induced cell death via necrosis rather than apoptosis pathway. Necrosis seems to be the dominant signaling pathway in mercury-induced sperm death, and it may be due to major damage to ultra-structures of sperm cells.

Since ultra-structural damage in sperm cells was observed even after 15 min of exposure to mercury, therefore, changes in motility and kinematic patterns, and necrosis in sperm cells may be due to abrupt stoppage of the functional machinery of sperm cell, especially mitochondria. Sperm DNA damage and apoptosis do not seem to be the main cause of instant sperm cell death as DNA damage was observed only after 3 h of exposure. DNA fragmentation coupled with severe ultrastructural damage seem to be the important mechanisms for delayed toxic effects including viability and altered functional dynamics of goat spermatozoa. Accordingly, based on our findings in the present study and those reported by us earlier^13^, we propose the possible mechanistic pathways of mercury-induced alterations in functional dynamics and death of goat spermatozoa (Fig. 12). However, possibility of involvement of certain other pathway(s) cannot be ruled out which may include cytochrome-c or TNF-α dependent and most likely direct activation of the caspase(s) that lead to necrosis.

## Conclusions

Exposure of goat spermatozoa to mercury resulted in instant damage to sperm plasma membrane, acrosome cap, mitochondrial sheath and microtubules of spermatozoa and thus cell death while DNA damage was observed only after 3 h. Mercury seems to primarily target sperm mitochondria and plasma membrane and thereby results in spontaneous cell death independent to apoptosis. Mercury-induced alterations in cellular integrity and functionality of sperm cells will affect the fertilizing competence of mercury-exposed animals. Therefore, to ensure the quality control of cryopreserved semen, it should be ensured that the semen of bucks does not contain mercury or other toxic metals.

## Supplementary Information

Supplementary Information.
