# Supplementary material for: Collapsed mitochondrial cristae in goat spermatozoa due to mercury result in lethality and compromised motility along with altered kinematic patterns
Source: Sci Rep. 2021 Jan 12;11:646. doi: 10.1038/s41598-020-80235-y (PMC7804962; doi:10.1038/s41598-020-80235-y)
Supplement: Supplementary file 1 — Supplementary Information. [file 41598_2020_80235_MOESM1_ESM.doc]

# Supplementary Data

# Collapsed mitochondrial cristae in goat spermatozoa due to mercury result in lethality and compromised motility along with altered kinematic patterns

**Bhawna Kushawaha; PhD^1^, ^4*^, Rajkumar Singh Yadav; PhD^2, 4^, Dilip Kumar Swain; PhD^3, 4^,** **Priyambada Kumari; PhD^1, 4^, Akhilesh Kumar; MSc^1, 4^, Brijesh Yadav; PhD^3, 4^, Mukul Anand; PhD^3, 4^, Sarvajeet Yadav; PhD^3, 4^, Dipty Singh; PhD ^5^ and Satish Kumar Garg; PhD ^2, 4^**

**College of Biotechnology^1^**

**Department of Veterinary Pharmacology and Toxicology^2^**

**Department of Veterinary Physiology^3^**

**U.P. Pandit Deen Dayal Upadhyaya Pashu Chikitsa Vigyan Vishwavidyalaya Evam Go Anusandhan Sansthan (DUVASU) ^4^**

**ICMR-National Institute for Research in Reproductive Health (NIRRH)^5^, Mumbai, India**

**Corresponding author^*^**

**Dr Bhawna Kushawaha (PhD)**

UP Pandit Deen Dayal Upadhyaya Pashu Chikitsa Vigyan Vishwavidyalaya

(Veterinary University, DUVASU), Mathura-281001, Uttar Pradesh- India

Current address- National Institute of Animal Biotechnology, INDIA

E-mail: bhawnarajput31jan@gmail.com

Phone: +918923776239


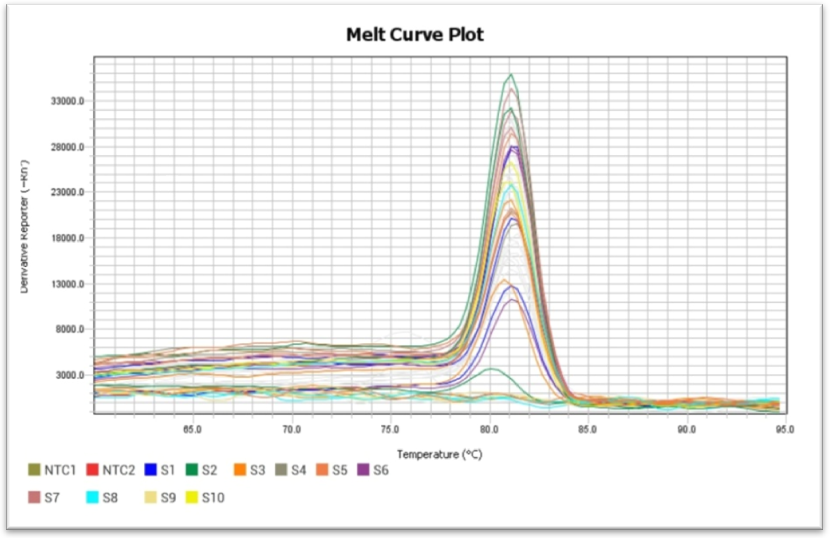
**Fig. 1: Melting curve of the Bcl-2 (where NTC 1 and 2= non template control, S1=PBS control, S2=0.031 µg/ml, S3-0.125 µg/ml, S4=0.25 µg/ml, S5=1.25 µg/ml of 15 min and S6= PBS control, S7=0.031 µg/ml, S8=0.125 µg/ml, S9=0.25 µg/ml, S10=1.25 µg/ml of 3 h)**


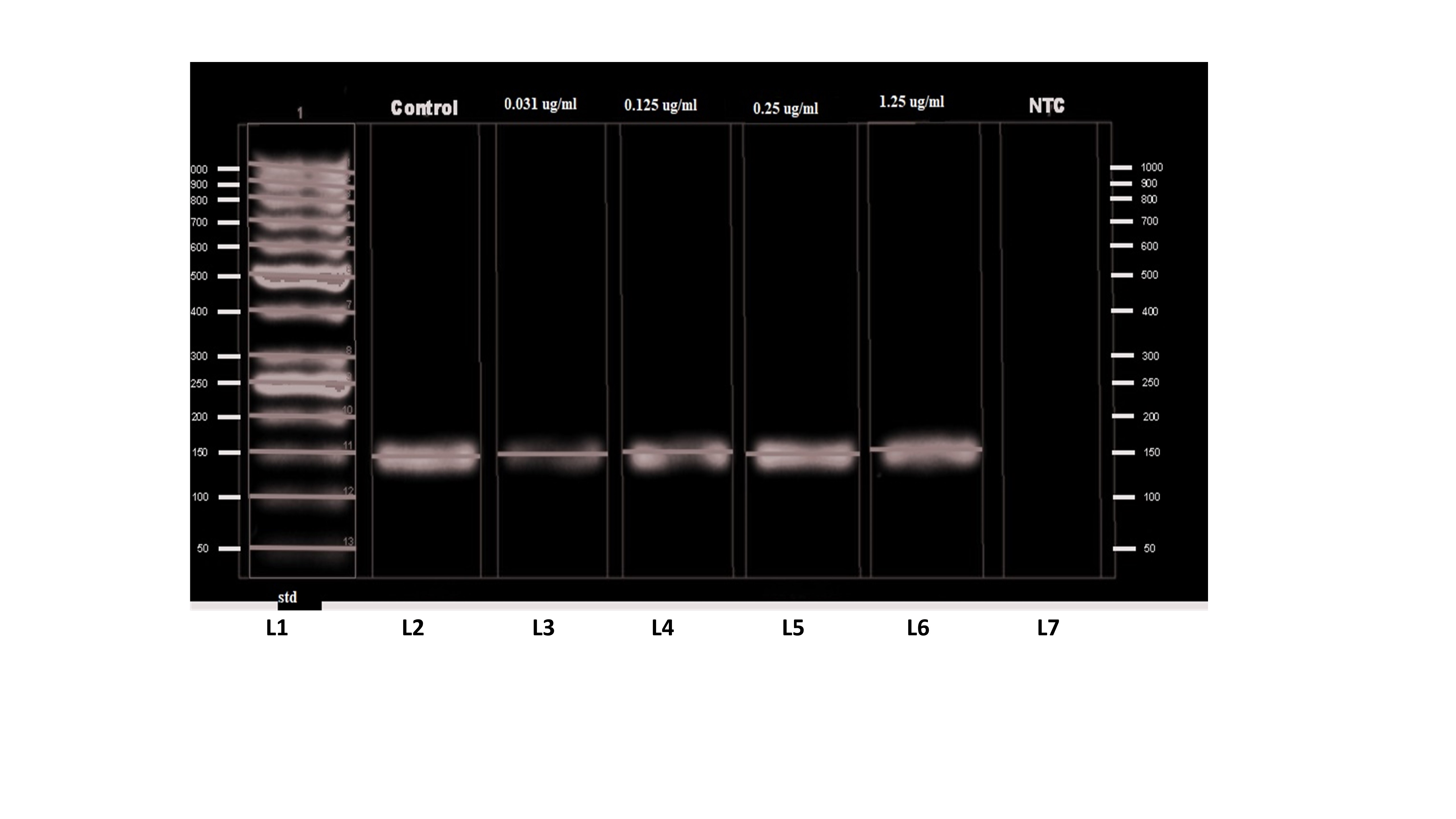


**Fig. 2: Amplification of Bcl-2 gene (143 bp) in the spermatozoa treated with different concentrations of mercuric chloride after 15 min and 3 h. Where, L1= Marker (50 bp), L2= PBS control, L3=0.031 µg/ml, L4=0.125 µg/ml, L5=0.25 µg/ml, 6=1.25 µg/ml HgCl_2_, L7-NTC**

**
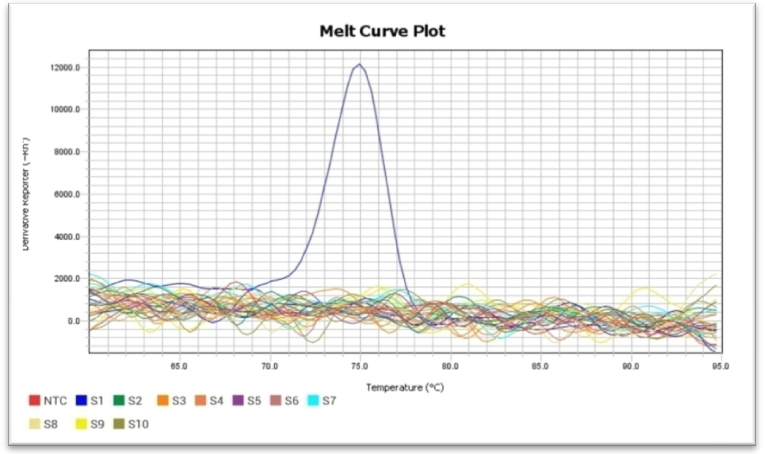
**

**Fig.3: Melting curve of Bax (where NTC 1= Non template control, S1=PBS control, S2=0.031 µg/ml, S3-0.125 µg/ml, S4=0.25 µg/ml, S5=1.25 µg/ml of 3 h and S6= PBS control, S7=0.031 µg/ml, S8=0.125 µg/ml, S9=0.25 µg/ml, S10=1.25 µg/ml of 15 min).**


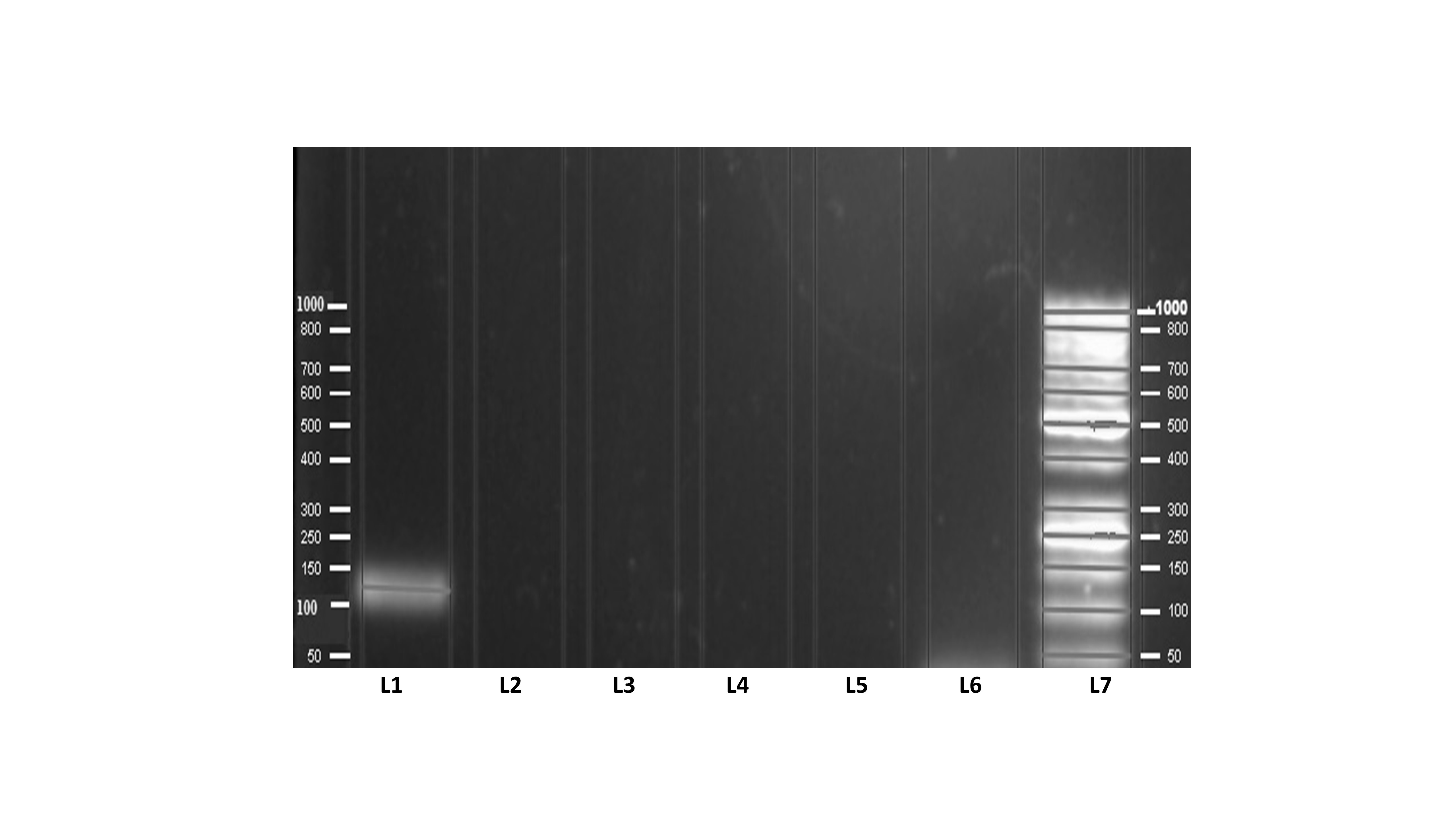


**Fig. 4: Amplification of Bax gene (101 bp) in the spermatozoa treated with different concentrations of mercuric chloride at 3 h. Where, L1= PBS control, L2=0.031 µg/ml, L3=0.125 µg/ml, L4=0.25 µg/ml, L5=1.25 µg/ml HgCl_2_, L6-NTC, L7-Marker (50 bp)**

**
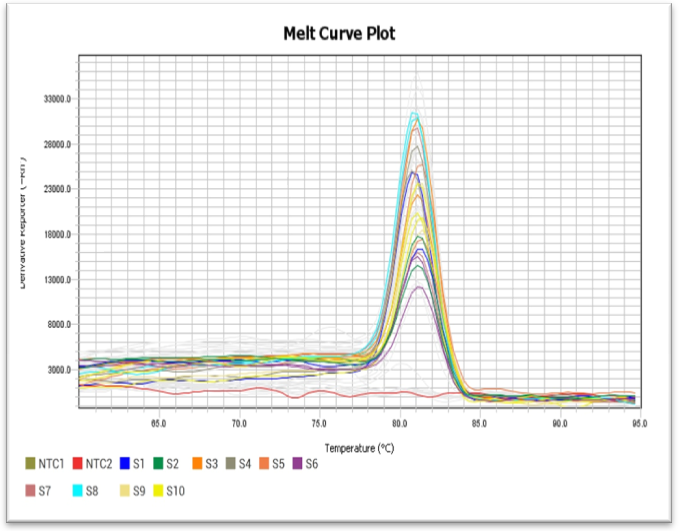
**

**Fig. 5: Melting curve of the β –actin (where NTC 1 and 2= non template control, S1=PBS control, S2=0.031 µg/ml, S3-0.125 µg/ml, S4=0.25 µg/ml, S5=1.25 µg/ml of 15 min and S6= PBS control, S7=0.031 µg/ml, S8=0.125 µg/ml, S9=0.25 µg/ml, S10=1.25 µg/ml of 3 h).**


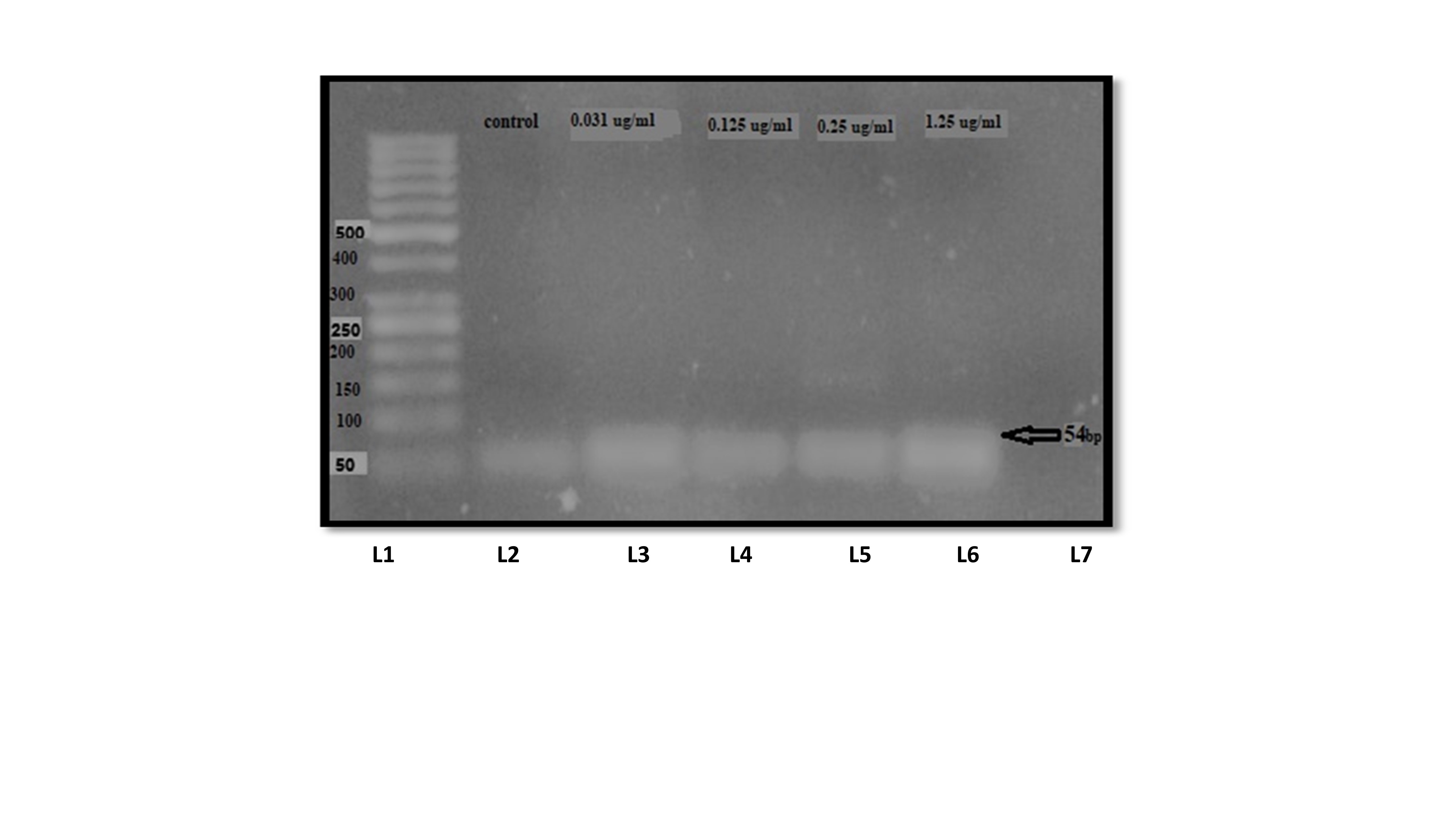


**Fig. 6: Amplification of** β**-actin** (**54 bp**) **gene in the spermatozoa treated with different concentrations of mercuric chloride at 15 min and 3 h. Where, L7-Marker (50 bp), L2= PBS control, L3=0.031 µg/ml, L4=0.125 µg/ml, L5=0.25 µg/ml, L6=1.25 µg/ml HgCl_2_, L7-NTC**
